# Phytochemical Characterization and Evaluation of Antioxidant and Tyrosinase Inhibitory Activities of *Verbascum wiedemannianum* Essential Oil and Methanolic Extract

**DOI:** 10.3390/molecules31111783

**Published:** 2026-05-22

**Authors:** Fatih Göger, Mehmet Tekin, Gülmira Özek, Süleyman Yur, Mevlüt Akdağ, Temel Özek

**Affiliations:** 1 Department of Pharmaceutical Botany, Faculty of Pharmacy, Afyonkarahisar Health Sciences University, 03030 Afyonkarahisar, Türkiye; 2 Department of Pharmaceutical Botany, Faculty of Pharmacy, Trakya University, 22030 Edirne, Türkiye; mtekin2280@gmail.com; 3 Department of Pharmacognosy, Faculty of Pharmacy, Anadolu University, 26470 Eskisehir, Türkiye; gozek@anadolu.edu.tr (G.Ö.); tozek@anadolu.edu.tr (T.Ö.); 4 Department of Pharmacognosy, Faculty of Pharmacy, Afyonkarahisar Health Sciences University, 03030 Afyonkarahisar, Türkiye; suleyman.yur@afsu.edu.tr; 5 Department of Pharmaceutical Chemistry, Faculty of Pharmacy, Afyonkarahisar Health Sciences University, 03030 Afyonkarahisar, Türkiye; mevlut.akdag@afsu.edu.tr; 6 Medicinal Plant, Drug and Scientific Research and Application Center (AUBIBAM), Anadolu University, 26470 Eskisehir, Türkiye

**Keywords:** *Verbascum wiedemannianum*, essential oil, extract, antioxidant, tyrosinase

## Abstract

*Verbascum* species have long been recognized for their medicinal properties; however, detailed studies on the endemic species *Verbascum wiedemannianum* Fisch. & C.A. Mey. remain limited. The purpose of this study is to evaluate the antioxidant and anti-tyrosinase activities of essential oil (EO) and methanol extract (ME) derived from *V. wiedemannianum*, an endemic species from Türkiye. The EO was obtained by hydrodistillation, and its chemical composition was characterized using GC-FID and GC/MS. The principal constituents of the EO were palmitic acid (27.3%), myristic acid (11.9%), 1-octadecanol (13.0%), and pentacosane (6.6%). LC-MS/MS analysis of the ME identified luteolin and chrysoeriol derivatives as the predominant compounds. The antioxidant potential of both the EO and ME was evaluated using three assay systems based on electron transfer reactions: the Folin–Ciocalteu reagent, the Trolox equivalent antioxidant capacity assay, and the cupric ion (Cu^2+^) reducing antioxidant capacity assay. The potential skin care effects of the EO and ME were further evaluated using a tyrosinase inhibition assay. Across all the assays, the ME consistently showed notable activities, whereas the activity of the EO was less clearly defined. These findings indicate that the ME of *V. wiedemannianum* contains bioactive compounds with potential applications in natural antioxidant and skin care formulations. Further studies are warranted to clarify its therapeutic uses.

## 1. Introduction

The genus *Verbascum* L, belonging to the *Scrophulariaceae* family, grows naturally in the Northern Hemisphere and is characterized by its bright yellow flowers. It comprises approximately 360 species worldwide, of which 200 are endemic to Türkiye [1,2,3,4,5]. Based on seed micromorphology, members of the *Verbascum* are divided into two subgenera, *Aulacospermae* and *Bothrospermae* [6]. All the *Verbascum* species in Türkiye are classified under the subgenus *Bothrospermae* [7]. Species of *Verbascum* are referred to as “sığırkuyruğu” in Anatolia and commonly known as mullein in Europe. Anatolia is regarded as the center of diversity for the genus *Verbascum*, characterized by a high level of endemism, approximately 80% [3,8]. *Verbascum* species are important for traditional and modern medicine due to their secondary metabolite content and long-standing ethnomedicinal applications. In Europe, the dried flowers of *V. thapsus* L., *V. densiflorum* Bertol., and *V. phlomoides* L. are collectively known as Flores Verbasci and used as expectorants in the treatment of cough [9]. The historical medicinal use of *Verbascum* species has led regulatory authorities to develop official monographs on the genus. The European Medicines Agency (EMA) and the European Pharmacopoeia have published monographs on Verbascum flos. According to the EMA, Verbascum flos is used for the relief of respiratory conditions, particularly dry coughs and colds, as well as for external applications to treat wounds, burns, and bedsores, and is associated with diuretic and antirheumatic effects [10]. Additionally, previous studies have reported its potential in managing diarrhea, sleeplessness, and anxiety [11] due to its rich content of various phytochemicals, including flavonoids, iridoid glycosides, saponins, tannins, and mucilage [12]. The antidiarrheal efficacy of *Verbascum* extracts stems from their polyphenolic compounds, particularly tannins; tannins form complexes with proteins in the intestinal mucosa, exerting a strong astringent effect [13]. This reduces tissue permeability and decreases liquid loss [14,15]. Flavanoids also provide further antidiarrheal and local anti-inflammatory benefits [13,16]. In addition to gastrointestinal relief, extracts derived from species such as *V. thapsus* have demonstrated significant sedative, pre-anesthetic, and anxiolytic activities in animal models, comparable to established central nervous system depressants like diazepam [17]. These neuropharmacological effects are mediated by the flavonoid profile of the genus, particularly apigenin and luteolin [17]. In previous studies, in silico models indicated that these flavonoids possessed strong binding affinities for critical neuroreceptors—such as adenosine A1 and A2a receptors and dopamine D4 receptors—which play a significant role in reducing neuronal excitability, inducing sedation, and promoting sleep [16,18]. The complex interaction between protein-binding tannins, mucilage polysaccharides with sedative properties, and neuromodulatory flavonoids underlies the clinical application of *Verbascum* species in the treatment of diarrhea, insomnia, and anxiety.

Recently, the European Food Safety Authority (EFSA) Panel on Additives and Products or Substances Used in Animal Feed has evaluated the efficacy of a tincture derived from *Verbascum thapsus* L. (great mullein tincture) as a sensory feed additive for all animal species [19].

In Anatolia, the traditional use of the genus is similar to its global application. Infusions prepared from the aerial parts of the three *Verbascum* species—*V. phlomoides*, *V. densiflorum*, and *V. thapsus*—are used as expectorants, an effect attributed to their high mucilage content [16,20].

In some regions of Anatolia, *V. orientale* L. flowers are used topically for the treatment of urogenital disorders and as an antipruritic agent after being boiled in milk [21]. Similar traditional applications have been reported for *V. pumilum* Boiss. & Heldr.; in this case, the steam generated by heating the plant in water is used in the treatment of anal fistulas [22]. The reported anti-inflammatory properties of the plant support its use in the management of hemorrhoids, inflammatory skin conditions, and rheumatic disorders [23]. *V. gypsicola* Vural & Aydoğdu exhibited substantial antibacterial activity, particularly against Gram-positive bacteria [24]. The use of *V. cheiranthifolium* Boiss. and *V. armenum* Boiss. & Kotschy ex Boiss. species in the treatment of intestinal parasites in animals has also been documented [25].

Additionally, previous studies have reported that *Verbascum* species exhibit a wide range of biological activities, including antimicrobial [26], antioxidant [27], wound-healing [28,29], antinociceptive, anti-inflammatory [22,30,31], cholinesterase inhibitory [32], cytotoxic, anticancer and antitumor effects [33,34,35], immunomodulatory [36], hepatoprotective [37], antihyperlipidemic [38], and antitussive activities [39]. *V. mucronatum* Lam. has been documented for its hemostatic, laxative, and wound-healing properties [22,40]. Tatlı and Akdemir conducted a comprehensive study on the *Verbascum* genus, reporting various secondary metabolites, including saponins, iridoid glycosides, phenylpropanoid glycosides, monoterpenoid glycosides, neolignan glycosides, flavonoids, steroids, alkaloids, and volatile components [41].

The broad biological activity and rich phytochemical composition of *Verbascum* species, particularly the endemic *V. wiedemannianum* in Türkiye—which has not been previously studied in detail—underscore the relevance of this study to both scientific and industrial fields.

*V. wiedemannianum* is an endemic species widely distributed in the northern and central regions of Türkiye [42]. Its distinctive red–purple flowers differentiate it from other *Verbascum* species. In some regions of Anatolia, this plant is used in the preparation of liqueur [43]. *V. wiedemannianum* has previously been reported to contain phenyl ethanoid glycosides (A–E) (specioside, verbascoside, and forsythoside), iridoid glycosides (aucubin, catalpol, angeloside, and ajugol), chlorinated iridoids (glutinoside and rehmaglutin D), flavonoid derivatives (luteolin), triterpenes (verbascosaponin, verbascosaponin A, and desrhamnosylverbascosaponin), ursan-type saponins (rosamutin and niga-ichigoside), and isoprenoid-derived aromatic compounds [44].

The use of *V. thapsus* as an herbal medicine in Europe further highlights the established relationship between the genus *Verbascum* and pharmacology and medicine. Reported biological activities, including antibacterial, antioxidant, and anti-inflammatory effects, provide a foundation for future research in plant chemistry, pharmacology, and biochemistry.

The traditional uses of this genus in Anatolia also offer valuable insights for future ethnopharmacological research. This study highlights the potential relevance of this endemic *Verbascum* species as a food preservative and nutraceutical. The ability of natural antioxidants to mitigate oxidative stress associated with chronic diseases, including cancer, cardiovascular diseases, and Alzheimer’s disease, underscores the potential impact of this research on human health. Previous studies have reported the antioxidant activity of the methanolic extract (ME) of *V. wiedemannianum* [27].

The importance of tyrosinase inhibitors in managing skin hyperpigmentation associated with excessive melanin accumulation, as well as the reported links between cosmetic and neurological disorders, underscores the potential relevance of this study to both the cosmetic industry and neurology. This study is particularly critical because it focuses on *V. wiedemannianum*, an endemic species to Türkiye, and explores natural alternatives to synthetic compounds based on the documented biological activity of the *Verbascum* species. Additionally, excessive melanin accumulation in skin disorders is linked to the activity of the tyrosinase enzyme, which catalyzes the conversion of 3,4-dihydroxyphenylalanine (l-DOPA) to dopaquinone, a key step in melanin biosynthesis. This biochemical pathway contributes to melanin production and has been implicated in both skin disorders and neurodegenerative diseases [45,46]. Given the growing interest in tyrosinase inhibitors for managing such conditions, this study may help identify new, natural compounds with potential applications in cosmetic and therapeutic contexts [47].

*Verbascum* species contain phenylethanoid glycosides, iridoid glycosides, flavonoids, and other secondary metabolites, which have the potential to control free radicals that cause cellular damage and regulate melanin synthesis [48]. Therefore, *V. wiedemannianum* was examined to determine whether it exhibits antioxidant and antimelanogenic (anti-tyrosinase) activities, particularly as a potential natural alternative to synthetic antioxidants and tyrosinase inhibitors. In the present study, the term was used specifically in reference to the inhibition of tyrosinase activity, which is a key enzyme involved in melanogenesis and is widely accepted as a preliminary indicator of antimelanogenic potential. Accordingly, this study was conducted to undertake a comprehensive phytochemical characterization of *V. wiedemannianum* species and evaluate its associated biological activities.

The main objective of this study was to characterize the phytochemical composition of the essential oil (EO) and extract of *Verbascum wiedemannianum* Fisch. & C.A. Mey., an endemic species native to Türkiye, and to subsequently evaluate their antioxidant and anti-tyrosinase potentials. The chemical composition of the plant was analyzed using GC-FID/MS and LC-MS/MS, while antioxidant capacity was assessed using Trolox equivalent antioxidant capacity (TEAC) and cupric ion reducing antioxidant capacity (CUPRAC) assays. Additionally, the tyrosinase inhibitory activity of this species is examined in greater detail, given the growing interest in identifying natural alternatives to synthetic antioxidants and in addressing melanin overproduction. Although several studies have reported the diverse biological activities of *Verbascum* species, the antioxidant activity of *V. wiedemannianum* has previously been examined by Tepe et al. [27].

We hypothesized that the methanol extract and essential oil of *V. wiedemannianum* contain distinct classes of bioactive phytochemicals that contribute to their antioxidant and tyrosinase inhibitory activities. We further hypothesized that the methanol extract, being rich in flavonoid derivatives such as luteolin and chrysoeriol glycosides, would exhibit stronger antioxidant and tyrosinase inhibitory effects than the essential oil. Finally, we proposed that the bioactive profile of this endemic species may support its potential use as a natural source for cosmetic and skin care-related applications, particularly in formulations targeting oxidative stress and tyrosinase-associated skin disorders.

The findings of this study provide scientific and practical insights into the broad potential applications of *V. wiedemannianum*. The results may help identify new biologically sourced antioxidants and tyrosinase inhibitors that could replace synthetic antioxidants. These findings may support the development of safer preservatives for the food industry, the formulation of skin-lightening and hyperpigmentation-reducing products in the cosmetics sector, and potentially new approaches to the treatment of neurodegenerative diseases.

## 2. Results

Hydrodistillation of the aerial parts of *V. wiedemannianum* yielded yellow oil with a specific odor. GC-FID and GC-MS techniques were used to determine the chemical content of the EO. Table 1 presents the identified compounds, their retention indices on the HP-Innowax FSC column, and their relative abundance.

**Figure 1:**
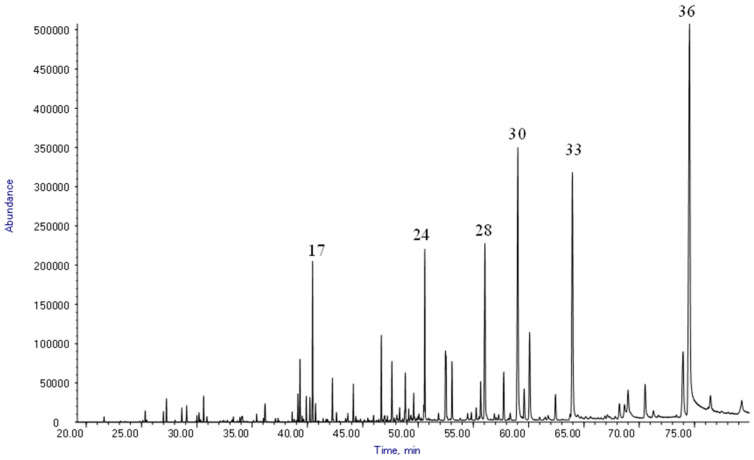
Chromatographic profile of *V. wiedemannianum* essential oil obtained on an HP-Innowax analytical polar column (peaks are numbered according to Table 1).

The EO of *V. wiedemannianum* exhibited a diverse profile of volatile constituents, which were classified as fatty acids and esters (42.2%), alkanes (18.2%), fatty alkohols (14.7%), oxygenated sesquiterpenes (3.4%), diterpenes (3.3%), C13-norisoprenoids (2.6%), oxygenated monoterpenes (0.9%), benzene derivatives (0.8%), and phenylpropanoids (1.5%). The chromatographic profile of *V. wiedemannianum* EO obtained using an analytical polar column is shown in Figure 1.

**Figure 2:**
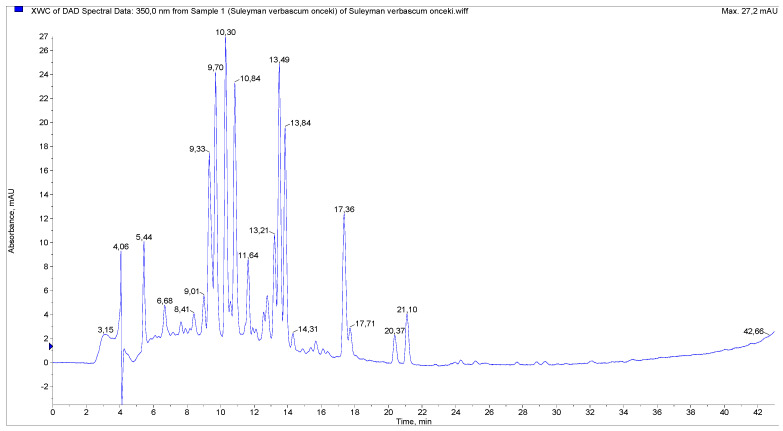
LC chromatogram of the *Verbascum* extract (350 nm).

Fifteen compounds were detected and monitored using LC-MS/MS, including 12 flavonoids, two phenylethanoid glycosides, and one unidentified constituent. The chromatographic profile of the extract is presented in Figure 2.

**Figure 3:**
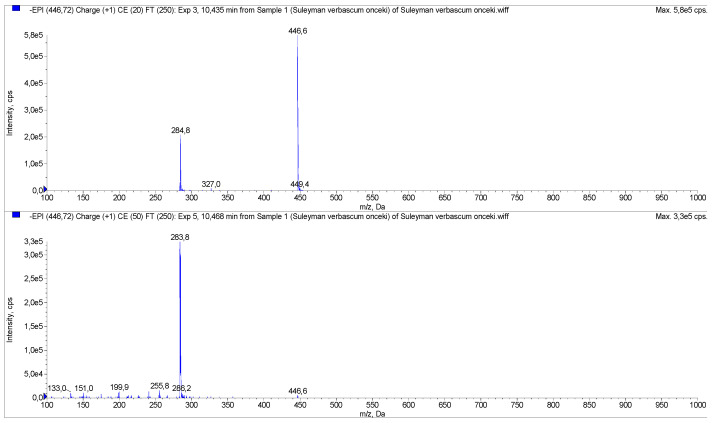
Mass spectrum of luteolin glucoside.

**Figure 4:**
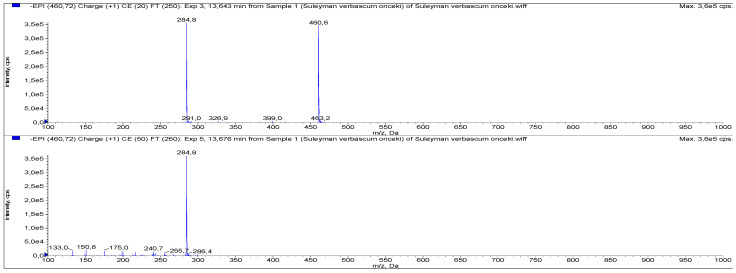
Mass spectrum of luteolin glucuronide.

Molecular ions, fragments observed in MS/MS, and the corresponding collision energies are presented in Table 2. The LC-MS analysis revealed that luteolin derivatives were the main compounds of the extract. Chrysoeriol (luteolin 3′-methyl ether) and its derivatives were also identified as major compounds. The mass spectra of luteolin glucoside and luteolin glucuronide are shown in Figure 3 and Figure 4 respectively.

The TPC and total flavonoid content of the ME of *V. wiedemannianum* are summarized in Table 3. The antioxidant activities of the EO and extract of *V. wiedemannianum* were evaluated using several assays. As presented in Table 3, the extract of *V. wiedemannianum* exhibited measurable activity in both test systems, with 1.0 mM Trolox equivalents in the TEAC assay and 36.0 mg/g BHT equivalents in the CUPRAC assay. Conversely, the EO was less active than the extract.

The *V. wiedemannianum* extract showed tyrosinase inhibitory activity. The major compounds identified were luteolin and chryseriol. As suggested in the literature, phenolic compounds inhibit the tyrosinase enzyme to some degree [63,64,65,66,67]. Thus, the major compounds were examined for their in silico binding affinities and interactions.

Luteolin and chryseriol are phenolic compounds (flavone derivatives) that differ only by an additional methyl group on chryseriol. According to our in silico studies, these two compounds showed similar binding affinities and binding modes. The binding affinity was −7.5 kcal/mol for both compounds.

**Figure 5:**
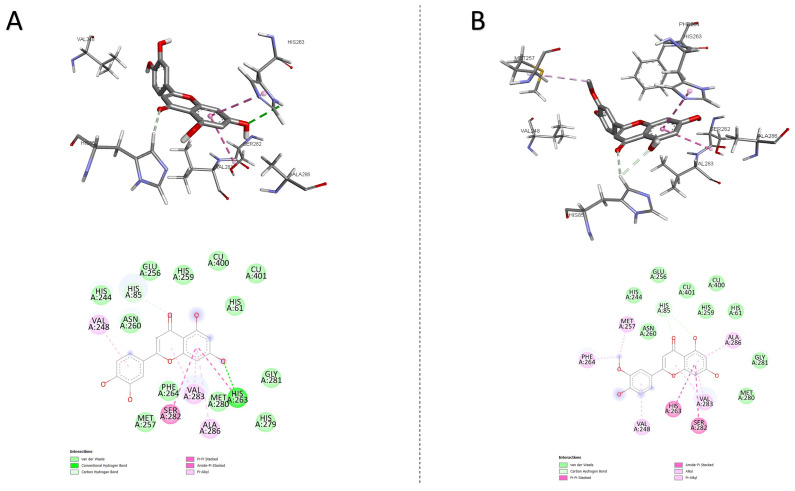
(**A**) Luteolin and its 3D and 2D interactions with the active site residues of the tyrosinase enzyme (PDB ID: 2Y9X). (**B**) Chryseriol and its 3D and 2D interactions with the active site residues of the tyrosinase enzyme (PDB ID: 2Y9X).

**Figure 6:**
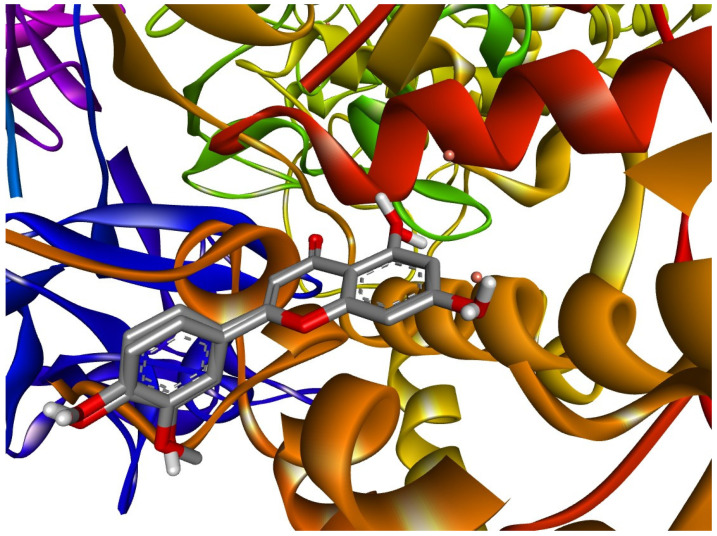
Superimposed docking poses of luteolin and chryseriol at the active site of the tyrosinase enzyme.

For luteolin, two hydrogen bond interactions were observed involving His263 and His85. A π-π stacking interaction between His263 and luteolin was also observed. Similarly, a π-π stacking interaction between chryseriol and His263 was observed for chryseriol. Recent in silico studies of tyrosinase inhibitors have also shown that the His263 residue stabilizes the enzyme-inhibitor combination at the catalytic site (PDB ID: 2Y9X) [68]. Two carbon–hydrogen bonds were observed for chryseriol through the His85 residue of the enzyme. Furthermore, both compounds were stabilized by similar van der Waals, alkyl, π-alkyl, and amide-π interactions, as shown in Figure 5. The docking poses of luteolin and chryseriol were superimposed on the active site of the tyrosinase enzyme, as shown in Figure 6.

## 3. Discussion

The novelty of the present work lies in providing the first comprehensive evaluation of both the essential oil and methanol extract of *V. wiedemannianum* with respect to their chemical composition, antioxidant potential, and tyrosinase inhibitory activity. Although several species of the genus *Verbascum* have been investigated previously, detailed phytochemical and bioactivity studies on this endemic species remain very limited. In particular, no previous study has comparatively characterized the volatile and phenolic profiles of this species using GC-MS and LC-MS/MS together with antioxidant and tyrosinase inhibition assays.

The rationale of the study is based on the growing interest in identifying plant-derived natural antioxidants and tyrosinase inhibitors for potential cosmetic and skin care applications. Since *Verbascum* species are known to contain flavonoids and other bioactive metabolites associated with antioxidant properties, we aimed to investigate whether *V. wiedemannianum* could represent a promising natural source of such compounds. The study was therefore designed to establish a relationship between the phytochemical composition of the extracts and their observed biological activities, thereby providing a clearer conceptual framework for the investigation.

Palmitic acid (27.3%), myristic acid (11.9%), 1-octadecanol (13.0%), and pentacosane (6.6%) were identified as the main constituents of EO. A literature search revealed a report by [69] describing the chemical diversity of volatiles obtained from the flowers, leaves, and stems of *V. wiedemannianum* collected from Kop Mountain in Erzurum, Türkiye. In that study, (2*E*)-hexenal (33.2%), pentadecane (58.2%), hexadecanoic acid (24.6%), and tetracosane (18.3%) were reported as the main constituents of the flower, leaf, and stem oils, respectively.

These differences may be attributed to variations in plant parts and analytical column characteristics in the study. While Iskender et al. reported hydrocarbons at a much higher rate using a nonpolar HP-5 column, the use of a polar HP-Innowax column in this study enabled high-resolution separation of fatty acids and alcohols. Overall, the results are consistent with previous findings, revealing the richness of *V. wiedemannianum* EO in non-terpenoid compounds, such as alkanes, aldehydes, alcohols, and fatty acids, rather than terpenoids.

In general, limited information is available on the volatile compounds of *Verbascum*. Previous reports indicate that the volatiles of *Verbascum* species are mostly composed of alkane series compounds. For example, 6,10,14-trimethyl-2-pentadecanone (14.3%) was identified in *V. thapsus* L., while 1-octen-3-ol (22.5%) and nonanal (9.0%) were found in *V. undulatum* Lam. [26,70]. In *V. creticum*, 1-octen-3-ol (23.9%), cis-3-hexen-1-ol (9.4%), phenylethanal (4.6%), and 2-methyl-benzofurane (4.6%) were identified as the main components [71]. Additionally, hexadecanoic acid (28.9%) and linoleic acid (7.3%) have been reported in *V. sinuatum* [72]. During hydrodistillation, not only highly volatile monoterpenes and sesquiterpenes but also certain less volatile lipophilic constituents, including long-chain fatty acids and related compounds, may co-distill and become part of the recovered oil fraction. Therefore, the detection of such constituents does not necessarily indicate contamination or improper extraction, but rather reflects the chemical complexity of the distillate obtained under these extraction conditions. Similar findings have been documented in numerous studies on essential oils from various plant taxa. In the literature there are reports about the fatty acids in the essential oil composition of *Verbascum* species [43,69].

Verbascoside and martinoside are phenylethanoyl glycosides previously identified in *V. wiedemannianum* [41,54] and previously reported for *V. wiedemannianum* [44]; however, as far as we know, luteolin glucoside (Figure 3) and luteolin glucuronide (Figure 4) are reported for the first time in this study. Apigenin and its derivatives (apigenin glucoside and apigenin pentoside), along with chrysoeriol and chrysoeriol glucoside, are known secondary metabolites of the *Verbascum* genus [41], but they have been identified in *V. wiedemannianum* for the first time. Rutin is also reported for the first time in this plant. Additionally, chrysoeriol glucuronide is reported here for the first time in the *Verbascum* genus.

The biological activities of EO and extracts of *Verbascum* species are well documented [73]. To identify novel classes of natural products with biological activity, the EO and ME of *V. wiedemannianum* were evaluated for antioxidant and anti-tyrosinase properties. Antioxidant activity was evaluated using free radical scavenging and reducing power assays. Anti-tyrosinase activity was evaluated by inhibiting tyrosinase activity during the oxidation of l-DOPA.

Within the scope of this study, the TPC and total flavonoid content were measured in the ME of *V. wiedemannianum*. Phenolic compounds in plants exhibit high antioxidant activity. When consumed by humans as food or used as food preservatives, they neutralize free radicals and prevent oxidative damage in their environment. Many diseases can be prevented by protecting against oxidative stress in the human body [13]. Numerous studies have reported that measurements of polyphenols in plants have become important tools for evaluating their relevance to human health [74,75,76].

It is known that antioxidants can act as free radical scavengers (e.g., DPPH^•^ and ABTS^•+^ scavenging assays), reducing activity (e.g., cupric ion reduction), or hydrogen atom donation [77,78]. Given that antioxidant activity may vary across assay systems, evaluating across multiple test methods is necessary to obtain a more comprehensive assessment of antioxidant properties.

Earlier, the ME of *V. wiedemannianum* and its phenylethanoid glycosides—wiedemannioside A–C, acteoside, martynoside, echinacoside, and leukoseptoside B—were screened for possible in vitro antioxidant activity using two complementary test systems: the DPPH free radical scavenging assay (by bioautography and spectrophotometry) and the β-carotene/linoleic acid test system. In the first system, *V. wiedemannianum* extract exhibited insignificant antioxidant activity. The compounds showed scavenging activity against the DPPH radical in TLC autographic assays. In the β-carotene/linoleic acid test system, *V. wiedemannianum* exhibited antioxidant activity [79]. Tepe et al. [27] reported on *V. wiedemannianum* ME tested with the DPPH free radical scavenging and β-carotene/linoleic acid systems.

The results of this study showed that the EO of *V. viedemannianum* exhibited weak antioxidant activity in the TEAC and CUPRAC assays, whereas the ME showed moderate activity. In the enzymatic assay, the extract showed inhibitory activity against the tyrosinase, whereas the EO was inactive. This study is the first in which the oil and extract of *V. viedemannianum* were comparatively evaluated for antioxidant and antityrosinase properties. The biological activity of the ME is due to the high abundance of phenolic compounds with flavonoid structures. Literature evidence indicates that flavonoids found in *Verbascum* species exhibit high antioxidant activity [80]. Studies have shown that tyrosinase inhibitory activity is high in flavonoids containing 4′C−OH and 5′C−OH [81]. Luteolin and its derivatives constitute a large proportion of the flavonoids in our ME, which fulfill this structural condition.

Molecular docking studies were performed to support the experimental findings. Luteolin and chryseriol, determined as major constituents of the studied extract, were chosen for in silico studies. The results exhibited that both compounds showed similar binding modes and comparable predicted binding affinities within the tyrosinase active site. This similarity is consistent with their closely related structures, differing only by a minor methyl substitution, suggesting that both compounds may contribute similarly to the observed biological activity.

In this study, the aerial parts of *V. wiedemannianum* were examined for their volatile composition, TPC and total flavonoid content, and antioxidant and antityrosinase activities. Given the longstanding traditional use of *Verbascum* species for various diseases, further research on these species is warranted.

This work shows *V. wiedemannianum*’s biological potential; however, it has limitations that should be explored in future research. The current study only includes in vitro and in silico molecular binding simulations. Thus, additional in vivo evaluations and clinical trials are needed to confirm the results. The essential oil’s weak antioxidant and tyrosinase-inhibiting activities, compared to the methanol extract, suggest that this endemic species’ industrial and pharmaceutical potential is primarily associated with its polar fractions, particularly flavonoids.

## 4. Materials and Methods

### 4.1. Plant Material

Plant material of *V. wiedemannianum* was collected from the following locality: Sivas, Hafik district, on the roadside leading to Durulmuş village, at the flowering stage. At 1320 m (9 June 2014), Dr. M. Tekin performed the collection and botanical identification of the species. A voucher specimen was deposited at the Herbarium of Cumhuriyet University, Faculty of Science, and assigned the voucher code M. Tekin 1579.

### 4.2. Chemicals and Reagents

The following chemicals were used in this study: *n*-hexane, methanol, ethanol, and dimethyl sulfoxide (DMSO). They were obtained from Sigma-Aldrich (St. Louis, MO, USA). Gallic acid and quercetin were purchased from Merck (Darmstadt, Germany), while a C_8_–C_40_ *n*-alkane standard solution was obtained from Fluka (Buchs, Switzerland). Tyrosinase from mushroom (EC 1.14.18.1), l-DOPA, and kojic acid were purchased from Sigma (St. Louis, MO, USA). Additional reagents included aluminum chloride, butylated hydroxytoluene (BHT), neocouproine (Nc), ammonium acetate, copper chloride, potassium persulfate, and (±)-6-hydroxy-2,5,7,8-tetramethylchromane-2-carboxylic acid (Trolox), all obtained from Sigma (Darmstadt, Germany). All the solvents used were of analytical grade.

### 4.3. Instruments

Gas chromatographic analyses were performed using an Agilent 5975 GC-MSD system (Agilent Technologies, Santa Clara, CA, USA) and a capillary gas chromatograph (Agilent 6890N GC system; SEM Ltd., Istanbul, Türkiye). LC-MS/MS studies were performed in a Shimadzu 20A HPLC system (Kyoto, Japan) coupled to an Applied Biosystems 3200 Q-Trap LC-MS/MS instrument (Concord, ON, Canada). Absorbance values were recorded using a microplate reader (ELISA system; Biotek PowerWave XS, Shoreline, WA, USA). Sample aliquots were dispensed into microplate wells using an Eppendorf^®^ Xplorer^®^ 12-channel pipettor (10–300 µL). A 96-deep-well, round-bottom polypropylene microplate (2.2 mL capacity) and a 96-well flat-bottom polystyrene microplate (nonsterile; Greiner, Frickenhausen, Germany) were purchased from Sigma-Aldrich (St. Louis, MO, USA).

### 4.4. Essential Oil Isolation

Aerial parts of *V. wiedemannianum* were hydrodistilled in a Clevenger apparatus for 3 h to obtain EO [82]. The resulting EO was dried over anhydrous sodium sulfate and stored in sealed vials at 4 °C until GC-FID, GC/MS analyses, and biological activity assays were performed. The EO was dissolved in *n*-hexane (10% *v*/*v*) for analysis of the chromatographic composition.

### 4.5. Extract Preparation

The plant material was extracted with methanol using a maceration method assisted by agitation at room temperature for 24 h, with a drug-to-solvent ratio of 1:10. The resulting extract was dried under vacuum.

### 4.6. GC-MS Analysis

The GC-MS analysis was performed using an Agilent 5975 GC-MSD system (Agilent Technologies, USA; SEM Ltd., Istanbul, Türkiye) equipped with an HP-Innowax FSC capillary column (60 m × 0.25 mm i.d., film thickness 0.25 µm; Agilent, Santa Clara, CA, USA). The carrier gas flow rate was set at 0.8 mL/min. The oven temperature was initially maintained at 60 °C for 10 min, then increased at 4 °C/min to 220 °C and held for 10 min. Subsequently, the temperature was further increased at 1 °C/min to 240 °C and held for 35 min. Total run time was 115 min. The oil sample was analyzed using a split ratio of 40:1. Mass spectra were recorded at 70 eV, and data were acquired over a mass range of *m*/*z* 35–450 amu.

### 4.7. GC-FID Analysis

The GC-FID analysis was conducted using a capillary GC (Agilent 6890N GC system; SEM Ltd., Istanbul, Türkiye) with the same chromatographic column and operating conditions as previously described. The GC injection port and FID detector temperatures were 250 °C and 280 °C, respectively, while the interface temperature was 280 °C. From the FID chromatograms, the relative percentage compositions of the separated compounds were calculated. Identification and quantification of individual compounds were performed using previously published literature data [83].

### 4.8. LC-MS/MS Analysis

LC-MS/MS analysis was performed using an Absciex 3200 Q trap MS/MS detector. Experiments were performed with a Shimadzu 20A HPLC system (Kyoto, Japan) coupled to an Applied Biosystems 3200 QTrap LC-MS/MS instrument equipped with an ESI ion source, which was used in the negative ionization mode. Separations were performed on a GL Science Intersil ODS 250 × 4.6 mm, i.d., 5 µm particle size, octadecyl silica gel analytical column operating at 40º C at a flow rate of 0.7 mL/min. Detection was carried out with a PDA detector. Elution was carried out using a binary gradient of the solvent mixture Acetonitrile:Water:Formic acid (10:89:1, *v*/*v*/*v*) (solvent A) and Acetonitrile:Water:Formic acid (89:10:1, *v*/*v*/*v*) (solvent B). The composition of B was increased from 10% to 100% in 40 min. For data acquisition and analysis, the Analyst 1.6 software was used. For enhanced mass scan (EMS), the MS was operated in the mass range of 100–1000 amu. Enhanced product ion spectra were measured from *m*/*z* 100 up to *m*/*z* 1000. Nitrogen was used as the collision gas, and the collision energy was set at 30. The parameters were as follows: Collusion Energy Spread (CES)—0; Declustering Potential (DP)—20; Entrance Potential (EP)—10; Curtain gas (CUR)—20; Gas Source 1 (GS1)—50; Gas Source 2 (GS2)—50; CAD—medium; Ihe—on and temperature (TEM)—600. For the IDA experiment, the criteria were arranged for ions greater than 100,000 *m*/*z* and smaller than 1000 *m*/*z* and excluded former target ions after 3.0 occurrence(s) for 3.000 s [45].

### 4.9. Total Phenolic Content

**Figure 7:**
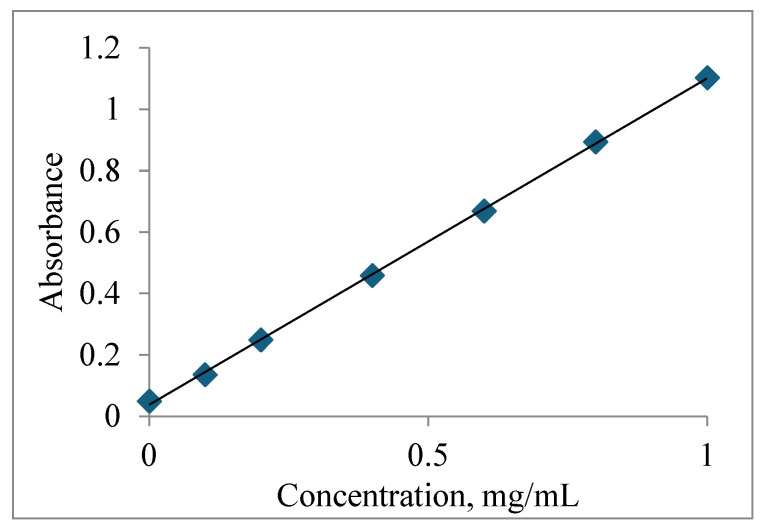
Concentration–response curve showing absorbance at 760 nm for the reduced FCR as a function of gallic acid standard concentration.

The total phenolic content of the extract was determined according to the method previously reported by Singleton [84]. Briefly, 50 μL of the sample (dissolved in methanol), 3.9 mL of pure water, and 250 µL of Folin–Ciocalteau reagent (FCR) were mixed and incubated in the dark for 8 min. Subsequently, 750 µL of sodium carbonate solution (20%, aqueous) was added, and the mixture was further incubated in the dark for 2 h. The absorbance was recorded at 760 nm. Calibration standards (1.0 mg/mL) were prepared in methanol to construct the calibration curve (Figure 7). The results were obtained from calculations using the regression equation (*y* = 1.0632x + 0.0377; *r^2^* = 0.9997). All the analyses were repeated three times, and the results were expressed as gallic acid equivalents (GAE).

### 4.10. Total Flavonoid Content

**Figure 8:**
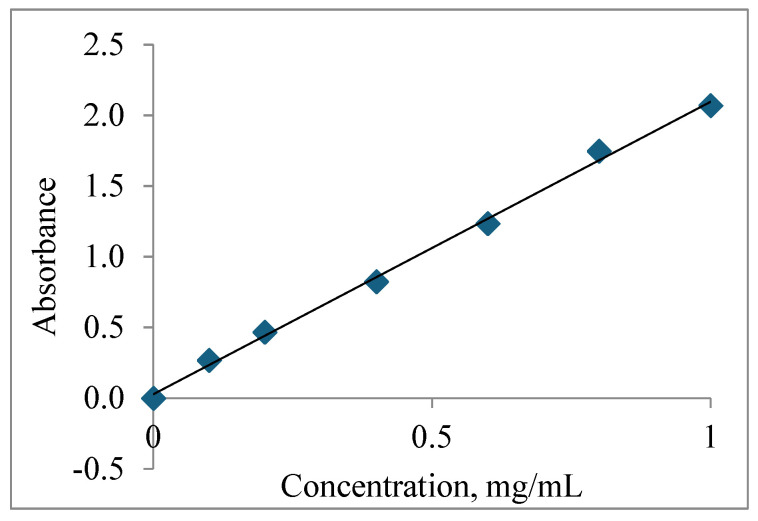
Concentration–response curve showing absorbance at 415 nm of the flavonoid–AlCl_3_ complex as a function of quercetin standard concentration.

The total flavonoid content of the extract was determined spectrophotometrically using AlCl_3_ according to Miliauskas et al., with slight modifications [85]. Briefly, 50 µL of the sample solvent (in methanol), 50 µL of AlCl_3_ solution (20 g/L), and 1.15 mL of absolute ethanol were mixed in a test tube, vortexed, and allowed to stand in the dark for 40 min. For the blank solution, AlCl_3_ was replaced with two drops of 15% acetic acid and 1.2 mL of absolute ethanol. The absorbance of the reaction mixture was measured at 415 nm using a UV–visible spectrophotometer (UV-PharmaSpec 1700, Shimadzu, Kyoto, Japan). A series of quercetin standard solutions (0.1–1.0 mg/mL) was prepared to construct the calibration curve (Figure 8). Quantification was performed using the regression equation (*y* = 2.0679x + 0.0281; *r^2^* = 0.9974). All the experiments were conducted in triplicate, and the results were expressed as mean quercetin equivalents (QE) ± standard error of the mean (SEM).

### 4.11. Trolox Equivalent Antioxidant Capacity Assay

The Trolox equivalent antioxidant capacity (TEAC) assay was performed according to previously reported procedures [86,87,88] with slight modifications. In this assay, the ability of the extracts to scavenge the ABTS radical cation was evaluated by comparing them with Trolox, a vitamin E analog. Briefly, ABTS was converted to its radical cation form (ABTS^•+^) by reacting with sodium persulfate (2.45 mM) for 16 h at room temperature in the dark. The resulting ABTS^•+^ solution (1 mL) was then diluted with ethanol to obtain an absorbance of 0.7–0.8 at 734 nm.

Trolox standard solutions (0.125–3.0 mM), EO (10 mg/mL), extract (10 mg/mL), and BHT (1 mg/mL) were prepared in absolute ethanol. The reaction mixture contained 10 µL of sample (EO, extract, or standard) and 990 µL of ABTS^•+^ solution. For the blank, ethanol was added instead of the sample. After 30 min of incubation, absorbance was measured at 734 nm using a UV–visible spectrophotometer (UV-PharmaSpec 1700, Shimadzu). All the experiments were performed in triplicate. The percentage of ABTS^•+^ scavenging activity was calculated using the following equation:

$$\%scavenging=\left[\left({Abs}_{{ABTS}^{\cdot +}}-{Abs}_{sample}^{30}/{Abs}_{{ABTS}^{\cdot +}}\right)\right]\times 100$$

**Figure 9:**
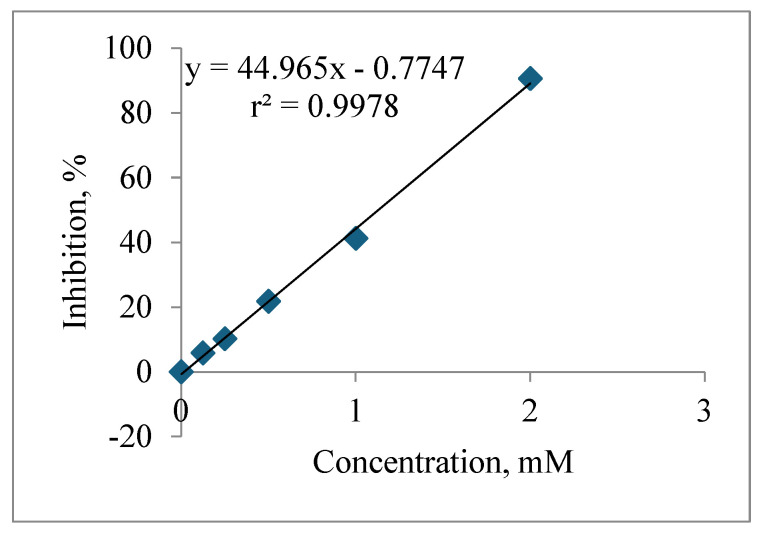
Concentration–response curve showing absorbance at 734 nm of ABTS^•+^ as a function of Trolox standard concentration.

The reduction in ABTS^•+^, indicated by a decrease in the blue–green color intensity, was quantitatively determined using TEAC mM at 734 nm. A calibration curve showing the relationship between ABTS^•+^ inhibition and Trolox concentrations (mM) is shown in Figure 9.

### 4.12. Cupric Reducing Antioxidant Capacity Assay

**Figure 10:**
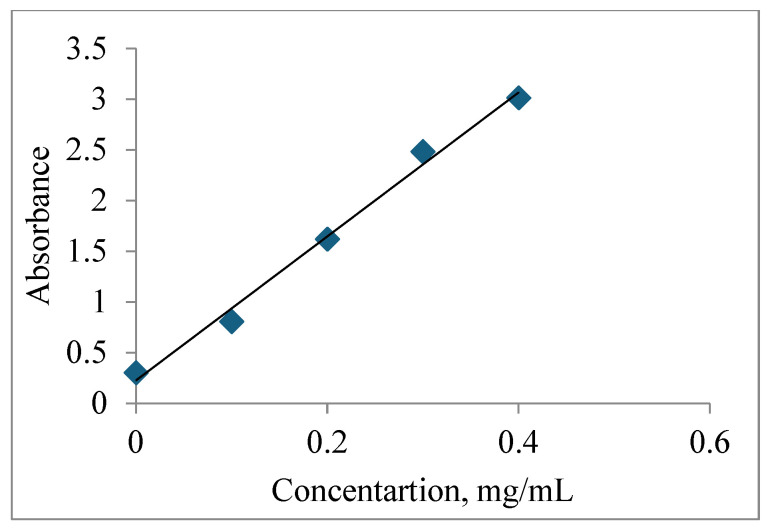
Concentration–response curve showing absorbance at 450 nm of reduced Cu^+^ as a function of BHT standard concentration.

The reducing power of essential oil and extract for copper ions was determined with the CUPRAC assay, which was performed with slight modification according to the method described by Apak et al. The assay evaluates the reducing capacity of the essential oil and extract toward copper(II) ions ([77]. Briefly, 55 μL of the samples (EO or extract) dissolved in methanol was added to 96-well flat-bottom plates, followed by 50 μL of CuCl_2_ solution (1.0 × 10^−2^ M), 50 μL of neocuproine solution (7.5 × 10^−3^ M), and 50 μL of NH_4_Ac buffer (pH 7.0, 1.0 M). The mixture was incubated in the dark at 25 °C for 30 min. For the control, a well containing 50 μL of methanol along with all the other reagents was used instead of the samples. Absorbance was measured at 450 nm using an ELISA microplate reader (Biotek Powerwave XS, Winooski, VT, USA). The results were obtained using various concentrations of standard BHT through the calibration curve (*y* = 7.099x + 0.2263; *r*^2^ = 0.9916) (Figure 10) and expressed as mg/g extract in BHT equivalents (BHTE).

### 4.13. Tyrosinase Inhibitory Activity

The antityrosinase potential of the samples was evaluated using a modified 96-well microplate assay based on the method described by Masuda et al. [89]. 1.0 mg/mL were prepared in 0.1 M phosphate buffer (pH 6.8) containing 20% DMSO. The assay consisted of four reaction groups: (A) control (120 μL buffer + 40 μL tyrosinase solution, 33.3 U/mL), (B) control blank (160 μL buffer), (C) sample (80 μL buffer + 40 μL tyrosinase + 40 μL sample solution), and (D) sample blank (120 μL buffer + 40 μL sample solution). Kojic acid (0.01–0.1 mg/mL) was used as the reference inhibitor. Following a 10 min preincubation at 23 °C, the enzymatic reaction was initiated by adding 40 μL of l-DOPA (2.5 mM). The mixture was further incubated for 15 min, and absorbance was measured at 475 nm using a BioTek PowerWave XS microplate reader. The resulting data from these four test groups (A, B, C, and D) were used to determine the percentage inhibition using the following formula:

$$Inh\%=\left\{\left[\left({Abs}_{A}-{Abs}_{B}\right)-\left({Abs}_{C}-{Abs}_{D}\right)\right]/({Abs}_{A}-{Abs}_{B})\right\}\times 100$$

**Figure 11:**
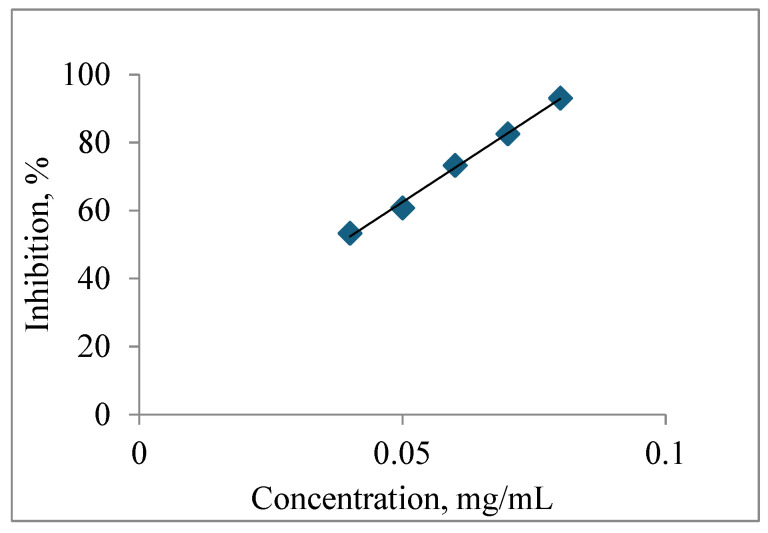
Kojic acid calibration curve.

The anti-tyrosinase activity of the essential oil and extract was shown as kojic acid equivalents (KAE, mg/g extract), calculated using a kojic acid standard curve (*y* = 1014.1x + 11.798, *r*^2^ = 0.9958), where *y* represents % inhibition and *x* represents concentration (mg/mL) in Figure 11. All the experiments were performed in triplicate, and the results were expressed as mean KAE values SEM.

### 4.14. Molecular Docking Studies

Molecular docking studies were performed to evaluate the in silico binding modes of luteolin and chrysoeriol. For these studies, the cocrystal structure of *A. bisporus* tyrosinase enzyme complexed with tropolone (PDB ID: 2Y9X) [90] was used, as the biological activity assays were conducted using mushroom tyrosinase. Chimera 1.17.3 was used in the molecular docking simulations. Following conventional ligand and protein preparation steps, docking studies were performed at the active site of the tyrosinase enzyme. The best-scoring poses of the ligands were selected for further investigation. Two-dimensional (2D) and three-dimensional (3D) interactions of chrysoeriol and luteolin were visualized using BİOVİA Discovery Studio (v21.1). Redocking of the co-crystallized tropolone ligand was performed to validate the docking protocol, yielding an RMSD value of 2.2 Å. The grid box was centered at X = 17.51, Y = 0.22, and Z = −93.91, with dimensions of 24 × 20 × 17 Å. Docking calculations were performed using the following search parameters: exhaustiveness = 8, energy_range = 3, and num_modes = 10. The binuclear copper center was retained in the active site during docking calculations; however, metal-centered interactions were not explicitly included in the scoring procedure.

### 4.15. Declaration of Generative AI Usage in Manuscript Preparation

In preparing this manuscript, the authors used Springer Nature Research Assistant AI’s manuscript advisor tool to obtain critical feedback on the draft. It is important to note that all the fundamental scientific components, including the study design, data analysis, and interpretation of findings, were executed entirely by the authors, without the involvement of artificial intelligence.

## 5. Conclusions

This study has provided a detailed elucidation of the phytochemical composition and biological activities of the endemic plant *Verbascum wiedemannianum*. Key findings revealed that the plant’s essential oil is rich in non-terpenoid compounds, while its methanol extract contains high levels of phenolic compounds that exhibit potent antioxidant and tyrosinase-inhibiting properties. To the best of our knowledge, luteolin glucoside and luteolin glucuronide have been identified in this species for the first time. Molecular docking studies of chrysoeriol and luteolin confirmed that they exhibit high binding affinity for the active site of the tyrosinase enzyme. While the study offers promising practical results for the development of food preservatives and skin-lightening cosmetic products, a significant limitation is that the evaluations are restricted to laboratory conditions (in vitro) and computer simulations (in silico). Therefore, clinical and in vivo studies are needed to validate the plant’s therapeutic efficacy.

## Figures and Tables

**Table 1:** Chemical composition of *Verbascum wiedemannianum* essential oil.

No	RRI ^a^	RRI ^b^	Compound	% ^c^	ID Method
1	1400	1400	Nonanal	0.4	d, e, and f
2	1446	1446	Dimethyl tetradecane	0.3	d, e, and f
3	1452	1446	1-Octen-3-ol	1.0	d, e, and f
4	1496	1474	2-Ethyl hexanol	0.6	d, e, and f
5	1500	1500	Pentadecane	0.6	d, e, and f
6	1541	1508	Benzaldehyde	0.4	d, e, and f
7	1553	1546	Linalool	0.9	d, e, and f
8	1655	1655	(*E*)-2-Decenal	0.2	d, e, and f
9	1663	1612	Phenylacetaldehyde	0.4	d, e, and f
10	1664	1662	Nonanol	0.1	d, e, and f
11	1706	1700	*α*-Terpineol	t	d, e, and f
12	1740	1709	Valencene	t	d, e, and f
13	1815	1791	2-Tridecanone	0.3	d, e, and f
14	1838	1838	(*E*)- *β*-Damascenone	0.8	e, f
15	1845	1838	(*E*)-Anethole	1.5	d, e, and f
16	1868	1843	(*E*)-Geranyl acetone	0.8	d, e, and f
17	1888	1882	1-Isobutyl 4-isopropyl 3-isopropyl-2,2-dimethyl succinate	3.1	e
18	1898	1894	1-(2-Hydroxy-1-methylethyl)-2,2-dimethylpropyl 2-methylpropanoate	0.4	f
19	1958	1914	(*E*)- *β* -Ionone	1.0	d, e, and f
20	2131	2131	Hexahydrofarnesyl acetone	1.8	d, e, and f
21	2179	2179	3,4-Dimethyl-5-pentylidene-2(5H)-furanone	1.5	e, f
22	2232	2200	*α*-Bisabolol	1.0	d, e, and f
23	2262	2224	Ethyl hexadecanoate (=*Ethyl palmitate*)	0.4	d, e, and f
24	2300	2300	Tricosane	4.6	d, e, and f
25	2387	2387	Cyclotetradecane	1.7	f
26	2390	2367	Farnesylacetone	1.6	e, f
27	2400	2400	Tetracosane	1.5	d, e, and f
28	2500	2500	Pentacosane	6.6	d, e, and f
28	2503	2503	Dodecanoic acid	0.9	d, e, and f
30	2607	2593	1-Octadecanol	13.0	d, e, and f
31	2615	2613	Ethyl linolenate	0.7	d, e, and f
32	2622	2622	Phytol	3.3	d, e, and f
33	2670	2624	Tetradecanoic acid (=*Myristic acid*)	11.9	d, e, and f
34	2843	-	14-Pentadecenoic acid	1.0	f
35	2900	2900	Nonacosane	4.3	d, e, and f
36	2931	2931	Hexadecanoic acid (=*Palmitic acid*)	27.3	d, e, and f
*Total*				*95.9*	
Oxygenated Monoterpenes				0.9	
Oxygenated Sesquiterpenes				3.4	
C13-norisoprenoids				2.6	
Fatty acids and esters				42.2	
Fatty alkohols				14.7	
Alkanes				18.2	
Benzene derivatives				0.8	
Diterpenes				3.3	
Phenylpropanoids				1.5	
Others				8.3	

RRI: Relative retention indices calculated against *n*-alkanes; %: calculated from FID data; t: trace (<0.1%). ^a^ Relative retention indices calculated against *n*-alkanes (C_9_–C_40_) on the HP-Innowax column. ^b^ Relative retention indices reported in the literature. ^c^ Percentage calculated from FID data. d Identification based on retention index of genuine compounds on the HP-Innowax column. e Identification based on mass spectral matching and retention data using the Başer Library. f Identification based on mass spectra matching from Adams (Adams [49], MassFinder [50], and WileyNIST libraries.

**Table 2:** LC-MS/MS analysis of the extract of *V. wiedemannianum*.

*t*_R_ (min)	[M-H]^−^ (*m*/*z*)	Ms/Ms (*m*/*z*)	Identification	Ref.
6.7	401	269, 161, 131	Apigenin pentoside	[51,52]
9.3	609	300, 271 and 255	Rutin	[53]
9.4	623	461, 315, 179, 161 and 135	Verbascoside	[41,54]
9.7	637	461, 285, 433, 355	Luteolin diglucuronide	[55,56]
10.3	447	285, 133	Luteolin glucoside	[57,58]
10.8	461	381, 327, 285, 175 and 151	Luteolin glucuronide	[41,58]
12.2	431	268	Apigenin glucoside	[41]
12.5	461	445, 299 and 283.	Chrysoeriol glucoside	[41,59]
13.2	475	299, 284	Chrysoeriol glucuronide	[60]
13.5	461	399, 285, 175, 151, 133	Luteolin glucuronide	[41]
13.8	651	505, 457, 410, 193, 175	Martynoside	[61]
17.4	285	175, 151 and 133	Luteolin	[44,57]
19.8	327	309, 291, 239, 229 and 211	Unknown (smilar to 3-hydroxy-4′,5,7-trimethoxyflavone)	
20.4	269	241, 225, 155, 117	Apigenin	[41]
21.1	299	284, 255, 227, 198 and 183	Chrysoeriol	[62]

**Table 3:** Biological activities of the essential oil and extract of *Verbascum wiedemannianum* (mean ± SEM).

	EO	ME	GA	BHT
Total phenol content ^a^	-	23.0 ± 0.1	-	-
Total flavonoid content ^b^	-	10.0 ± 0.1	-	-
ABTS^•+^ scavenging activity ^c^	0.33 ± 0.03	1.0 ± 0.02	2.9 ± 0.004	2.7 ± 0.055
Cupric reducing antioxidant capacity ^d^	27.0 ± 2.0	36.0 ± 1.0	-	-
Tyrosinase inhibition ^e^	NA	2.1 ± 0.6	-	-

^a^ Expressed as mg GAE/g_extract_. ^b^ Expressed as mg QE/g_extract_. ^c^ Expressed as TEAC mM. ^d^ Expressed as mg BHTE/g_extract_. ^e^ Expressed as mg KAE/g_extract_. NA: not active.

## Data Availability

The original contributions presented in this study are included in the article/Supplementary Materials. Further inquiries can be directed to the corresponding author.

## Supplementary Materials

The following supporting information can be downloaded at https://www.mdpi.com/article/10.3390/molecules31111783/s1. Individual LC-MS/MS spectra presented as Supplementary Material.

## Abbreviations

The following abbreviations are used in this manuscript:

ABTS•^+^	ABTS radical cation
BHT	Butylated hydroxytoluene
BHTE	BHT equivalents
CUPRAC	Cupric ion reducing antioxidant capacity
DMSO	Dimethyl sulfoxide
EFSA	European Food Safety Authority
EMA	European Medicines Agency
EO	Essential oil
FCR	Folin–Ciocalteau reagent
GAE	Gallic acid equivalents
GC-FID	Gas chromatography–flame ionization detector
GC-MS	Gas chromatography–mass spectrometry
KAE	Kojic acid equivalents
l-DOPA	3,4-dihydroxyphenylalanine
LC-MS/MS	Liquid chromatography–tandem mass spectrometry
ME	Methanol extract
Nc	Neocouproine
QE	Quercetin equivalents
RRI	Relative retention indices
SEM	Standard error of the mean
SOD	Superoxide dismutase
TEAC	Trolox equivalent antioxidant capacity
TPC	Total phenolic content
Trolox	(±)-6-hydroxy-2,5,7,8-tetramethylchromane-2-carboxylic acid

## References

[1] Heywood V. (1993). Flowering Plants of the World.

[2] Mabberley D.J. (2008). Mabberley’s Plant-Book.

[3] Erdogan M.K., Sever A., Gundogdu R., Toy Y., Gecibesler I.H., Yapar Y., Behcet L., Zengin G. (2024). *Verbascum gimgimense* an Endemic Turkish Plant: Evaluation of In Vitro Anticancer, Antioxidant, Enzyme Inhibitory Activities, and Phytochemical Profile. Cell Biochem. Funct..

[4] Ekim T., Verbascum L. (2000). Flora of Turkey and the East Aegean Islands.

[5] Huber-Morath A., Verbascum L. (1978). Flora of Turkey and the East Aegean Islands.

[6] Murberck S. (1933). Monographie Der Gattung Verbascum.

[7] Huber-Morath A. (1971). Die Türkishchen Verbasceen.

[8] Karaveliogullari F.A., Ocak A., Ekici M., Cabi E. (2009). *Verbascum eskisehirensis* sp. nov (Scrophulariaceae) from central Anatolia, Turkey. Nord. J. Bot..

[9] Grigorov M., Pavlovic D., Antic S.M., Kostov M.T., Ilic D. (2023). In Vitro Antimicrobial Activity of Different *Verbascum Niveum* Extracts. Acta Fac. Medicae Naiss..

[10] European Medicines Agency (2018). Assessment Report on Verbascum thapsus L., Verbascum densiflorum Bertol. (Verbascum thapsiforme Schrad.), Verbascum phlomoides L., Flos.

[11] Demirci S., Alp C., Aksit H., Ulutas Y., Altay A., Yeniçeri E., Köksal E., Yayli N. (2023). Isolation, characterization and anticancer activity of secondary metabolites from *Verbascum speciosum*. Chem. Biol. Drug Des..

[12] Taleb S., Saeedi M. (2021). The effect of the *Verbascum thapsus* on episiotomy wound healing in nulliparous women: A randomized controlled trial. BMC Complement. Med. Ther..

[13] Bravo L. (1998). Polyphenols: Chemistry, dietary sources, metabolism, and nutritional significance. Nutr. Rev..

[14] Song Y., Luo Y., Yu B., He J., Zheng P., Mao X., Huang Z., Luo J., Luo Y., Yan H. (2021). Tannic acid extracted from gallnut prevents post-weaning diarrhea and improves intestinal health of weaned piglets. Anim. Nutr..

[15] Yu J., Song Y., Yu B., He J., Zheng P., Mao X., Huang Z., Luo Y., Luo J., Yan H. (2020). Tannic acid prevents post-weaning diarrhea by improving intestinal barrier integrity and function in weaned piglets. J. Anim. Sci. Biotechnol..

[16] Blanco-Salas J., Hortigón-Vinagre M.P., Morales-Jadán D., Ruiz-Téllez T. (2021). Searching for Scientific Explanations for the Uses of Spanish Folk Medicine: A Review on the Case of Mullein (*Verbascum*, Scrophulariaceae). Biology.

[17] Rezaie A., Ebrahimi M., Issabeagloo E., Kumar M., Nazeri M., Rezaie S., Zakhireh S. (2012). Study of sedative, pre-anesthetic, and anti-anxiety effects of *Verbascum thapsus* L. extract compared with diazepam in rats. Adv. Biosci. Res..

[18] Phillis J.W. (2001). Adenosine A2A receptor ligands: Effects on neuronal excitability. Drug Dev. Res..

[19] Bampidis V., Azimonti G., de Lourdes Bastos M., Christensen H., Kouba M., Kos Durjava M., López-Alonso M., López Puente S., Marcon F., EFSA Panel on Additives and Products or Substances used in Animal Feed (FEEDAP) (2019). Safety and efficacy of a tincture derived from *Verbascum thapsus* L. when used as a sensory additive in feed for all animal species. EFSA J..

[20] Gupta A., Atkinson A.N., Pandey A.K., Bishayee A. (2022). Health-promoting and disease-mitigating potential of *Verbascum thapsus* L. (common mullein): A review. Phytother. Res..

[21] Sezik E., Yeşilada E., Honda G., Takaishi Y., Takeda Y., Tanaka T. (2001). Traditional medicine in Turkey X. Folk medicine in Central Anatolia. J. Ethnopharmacol..

[22] Akdemir Z., Kahraman Ç., Tatlı I.I., Akkol E.K., Süntar I., Keles H. (2011). Bioassay-guided isolation of anti-inflammatory, antinociceptive and wound healer glycosides from the flowers of *Verbascum mucronatum* Lam. J. Ethnopharmacol..

[23] Yılmaz M., Genç G.E. (2024). Overview of ethnobotanical, phytochemical and biological activity relations of *Verbascum* species in worldwide. Turk. J. Biodivers..

[24] Dulger B., Gonuz A. (2004). Antimicrobial activity of some endemic *Verbascum*, *Salvia*, and *Stachys* species. Pharm. Biol..

[25] Korkmaz M., Alpaslan Z. (2014). Ergan Dağı Erzincan-Türkiye’nın etnobotanik özellikleri. Bağbahçe Bilim Derg..

[26] Morteza-Semnani K., Saeedi M., Akbarzadeh M. (2012). Chemical Composition and Antimicrobial Activity of the Essential Oil of *Verbascum thapsus* L.. J. Essent. Oil Bear. Plants.

[27] Tepe B., Sokmen M., Akpulat H.A., Yumrutas O., Sokmen A. (2006). Screening of antioxidative properties of the methanolic extracts of *Pelargonium endlicherianum* Fenzl., *Verbascum wiedemannianum* Fisch. & Mey., *Sideritis libanotica* Labill. subsp. *linearis* (Bentham) Borm., *Centaurea mucronifera* DC. and *Hieracium cappadocicum* Freyn from Turkish flora. Food Chem..

[28] Sohrabi-Haghdost R.V.H., Safarmashaei S. (2011). Comparison of in-vivo wound healing activity of *Verbascum thapsus* flower extract with zinc oxide on experimental wound model in rabbits. Adv. Environ. Biol..

[29] Süntar I., Tatlı I.I., Akkol E., Keleş H., Kahraman Ç., Akdemir Z. (2010). An ethnopharmacological study on *Verbascum* species: From conventional wound healing use to scientific verification. J. Ethnopharmacol..

[30] Kupeli E., Tatli I.I., Akdemir Z.S., Yesilada E. (2007). Bioassay-guided isolation of anti-inflammatory and antinociceptive glycoterpenoids from the flowers of *Verbascum lasianthum* Boiss. ex Bentham. J. Ethnopharmacol..

[31] Tatli I., Akdemir Z.S., Yesilada E., Küpeli E. (2008). Anti-inflammatory and antinociceptive potential of major phenolics from *Verbascum salviifolium* Boiss. Z. Naturforsch. C.

[32] Kahraman C., Tatlı I., Orhan I.E., Akdemir Z.S. (2010). Cholinesterase inhibitory and antioxidant properties of *Verbascum mucronatum* Lam. and its secondary metabolites. Z. Naturforsch. C.

[33] Gałasiński W., Chlabicz J., Paszkiewicz-Gadek A., Marcinkiewicz C., Gindzieński A. (1995). The substances of plant origin that inhibit protein biosynthesis. Acta Pol. Pharm..

[34] Paszkiewicz-Gadek A., Grochowska K., Gałasiński W. (1990). Effect of the aqueous extract and saponin fraction from the flowers of *Verbascum thapsiforme* on protein biosynthesis in a rat liver ribosomal system. Phytother. Res..

[35] Turker A.U., Camper N. (2002). Biological activity of common mullein, a medicinal plant. J. Ethnopharmacol..

[36] Klimek B., Stepien H. (1994). P17 Effect of some constituents of Mullein (*Verbascum* sp.) on proliferation of Rat splenocytes in vitro. Eur. J. Pharm. Sci..

[37] Mahmoud S.M., Shahat A.A., Hammouda F.M. (2007). Phytochemical and biological studies on *Verbascum sinaiticum* growing in Egypt. Nat. Prod. Sci..

[38] Aboutabl E., Goneid M., Soliman S., Selim A. (1999). Analysis of certain plant polysaccharides and study their antihyperlipidemic activity. J. Pharm. Sci..

[39] Nosalova G., Sutovska M., Mokry J., Kardosova A. (2005). Efficacy of herbal substances according to cough reflex. Health.

[40] Baytop T. (1999). Türkiye’de Bitkiler ile Tedavi: Geçmişte ve Bugün.

[41] Tatli I., Akdemir Z.S. (2004). Chemical constituents of *Verbascum* L. species. FABAD J. Pharm. Sci..

[42] Tekın M., Yılmaz G. (2018). Anatomical and palynological studies on endemic *Verbascum weidemannianum* Fisch. & Mey. (Scrophulariaceae) in Turkey. Int. J. Agric. For. Life Sci..

[43] Boğa M., Ertaş A., Yılmaz M.A., Kızıl M., Çeken B., Haşimi N., Özden T.Y., Demirci S., Yener İ., Deveci Ö. (2016). UHPLC-ESI-MS/MS and GC-MS Analyses on Phenolic, Fatty Acid and Essential Oil of *Verbascum pinetorum* with Antioxidant, Anticholinesterase, Antimicrobial and DNA Damage Protection Effects. Iran. J. Pharm. Res..

[44] Abou Gazar H., Tasdemir D., Ireland C.M., Çalis I. (2003). Iridoids and triterpene saponins from *Verbascum wiedemannianum* (Scrophulariaceae). Biochem. Syst. Ecol..

[45] Haliloglu Y., Ozek T., Tekin M., Goger F., Baser K.H.C., Ozek G. (2017). Phytochemicals, antioxidant, and antityrosinase activities of *Achillea sivasica* Çelik and Akpulat. Int. J. Food Prop..

[46] Yur S., Tekin M., Goger F., Baser K.H.C., Ozek T., Ozek G. (2018). Composition and potential of *Tanacetum haussknechtii* Bornm. Grierson as antioxidant and inhibitor of acetylcholinesterase, tyrosinase, and *α*-amylase enzymes. Int. J. Food Prop..

[47] Hassan M., Shahzadi S., Kloczkowski A. (2023). Tyrosinase inhibitors naturally present in plants and synthetic modifications of these natural products as anti-melanogenic agents: A review. Molecules.

[48] Zengin G., Mahomoodally M.F., Sinan K.I., Sadeer N., Maggi F., Caprioli G., Angeloni S., Mollica A., Stefanucci A., Ak G. (2021). Evaluation of chemical constituents and biological properties of two endemic *Verbascum* species. Process Biochem..

[49] Adams R.P. (1995). Identification of Essential Oil Components by Gaz Chromatography/Mass Spectroscopy.

[50] Joulain D., König W.A., Hochmuth D.H. (2001). Library of MassFinder-4.

[51] Guimarães R., Barros L., Dueñas M., Carvalho A.M., Queiroz M.J.R.P., Santos-Buelga C., Ferreira I.C.F.R. (2013). Characterisation of phenolic compounds in wild fruits from Northeastern Portugal. Food Chem..

[52] Küçük S., Has M., Göger F., Özdemir F., İncesu Z. (2023). Determination of cytotoxic, antioxidant activities and LC/MS-MS profiles of three endemic *Verbascum* L. species. Eur. J. Life Sci..

[53] Gvazava L.N., Kikoladze V.S. (2012). Orobanchoside and flavonoids from *Verbascum phlomoides* and *V. densiflorum*. Chem. Nat. Compd..

[54] Çalis I., Gazar H.A., Bedir E., Khan I.A. Phenylethanoid glycosides with free radical scavenging properties from *Verbascum wiedemannianum*. Proceedings of the 3rd IUPAC International Conference on Biodiversity.

[55] Cvetkovikj I., Stefkov G., Acevska J., Stanoeva J.P., Karapandzova M., Stefova M., Dimitrovska A., Kulevanova S. (2013). Polyphenolic characterization and chromatographic methods for fast assessment of culinary *Salvia* species from South East Europe. J. Chromatogr. A.

[56] Serralheiro M.L., Guedes R., Fadel S.R., Bendif H. (2020). Data on identification of primary and secondary metabolites in aqueous extract of *Verbascum betonicifolium*. Data Brief.

[57] Sen B., Dosler S., Mericli A. (2015). Chemical Constituents and Antimicrobial Activity of *Verbascum lagurus*. Chem. Nat. Compd..

[58] Öztürk G., Ağalar H.G., Yildiz G., Göger F., Kirimer N. (2019). Biological activities and luteolin derivatives of *Verbascum eskisehirensis* Karavel., Ocak & Ekici. J. Res. Pharm..

[59] Tatli I.I., Takamatsu S., Khan I.A., Akdemir Z.S. (2007). Screening for free radical scavenging and cell aggregation inhibitory activities by secondary metabolites from Turkish *Verbascum* species. Z. Naturforsch. C.

[60] Kwak J.H., Kim H.J., Lee K.H., Kang S.C., Zee O.P. (2009). Antioxidative iridoid glycosides and phenolic compounds from *Veronica peregrina*. Arch. Pharmacal Res..

[61] Li C., Liu Y., Abdulla R., Aisa H.A., Suo Y. (2014). Characterization and identification of chemical components in *Neopicrorhiza scrphulariiflora* roots by liquid chromatography-electrospray ionization quadrupole time-of-flight tandem mass spectrometry. Anal. Methods.

[62] Huang W., Wu S.-B., Wang Y.-L., Guo Z.-Y., Kennelly E.J., Long C.-L. (2013). Chemical constituents from Striga asiatica and its chemotaxonomic study. Biochem. Syst. Ecol..

[63] Yazar M., Sevindik M., Polat A.O., Koçer O., Karatepe H.K., Uysal İ. (2024). General properties, biosynthesis, pharmacological properties, biological activities and daily uses of luteolin. Prospect. Pharm. Sci..

[64] Sabudak T., Demirkiran O., Ozturk M., Topcu G. (2013). Phenolic compounds from *Trifolium echinatum* Bieb. and investigation of their tyrosinase inhibitory and antioxidant activities. Phytochemistry.

[65] Tan X., Song Y.H., Park C., Lee K.-W., Kim J.Y., Kim D.W., Kim K.D., Lee K.W., Curtis-Long M.J., Park K.H. (2016). Highly potent tyrosinase inhibitor, neorauflavane from *Campylotropis hirtella* and inhibitory mechanism with molecular docking. Bioorg. Med. Chem..

[66] Ng L.-T., Ko H.-H., Lu T.-M. (2009). Potential antioxidants and tyrosinase inhibitors from synthetic polyphenolic deoxybenzoins. Bioorg. Med. Chem..

[67] Kim D.W., Woo H.S., Kim J.Y., Ryuk J.A., Park K.H., Ko B.S. (2016). Phenols displaying tyrosinase inhibition from *Humulus lupulus*. J. Enzym. Inhib. Med. Chem..

[68] Suryani S.D., Wiarni N.P., Syahroni M.A., Syafri S., Hamidi D. (2026). Exploring anti-tyrosinase and photoprotective activities of *Curcuma heyneana* (val.) and *Kaempferia galanga* (L.) essential oils: In vitro and in silico approaches. Prospect. Pharm. Sci..

[69] Iskender N.Y., Yayli N., Yildrim N., Cansu T.B., Terzioglu S. (2009). The volatile constituents of the flower, leaf, and stem of *Verbascum wiedemannianum* grown in Turkey. J. Oleo Sci..

[70] Melliou E., Magiatis P., Kalpoutzakis E., Tsitsa E. (2007). Composition of the Essential Oil of *Verbascum undulatum* from Greece. J. Essent. Oil Res..

[71] Vaglica A., Porrello A., Ilardi V., Bruno M. (2025). The essential oil chemical composition of a rare ethnopharmacoligical plant: *Verbascum creticum* (L.) Cav. Nat. Prod. Res..

[72] Mohammadhosseini M. (2025). The first report on the screening of the GC/MS profiles of the hydrodistilled essential oils and volatile components from the aerial parts of *Verbascum sinuatum* L. (Scrophulariaceae). Trends Phytochem. Res..

[73] Kahraman C., Akdemir Z., Tatli I. (2012). Promising cytotoxic activity profile, biological activities and phytochemical screening of *Verbascum* L. species. Med. Aromat. Plant Sci. Biotechnol..

[74] Borges Bubols G., da Rocha Vianna D., Medina-Remon A., von Poser G., Maria Lamuela-Raventos R., Lucia Eifler-Lima V., Cristina Garcia S. (2013). The antioxidant activity of coumarins and flavonoids. Mini Rev. Med. Chem..

[75] Hosu A., Cristea V.-M., Cimpoiu C. (2014). Analysis of total phenolic, flavonoids, anthocyanins and tannins content in Romanian red wines: Prediction of antioxidant activities and classification of wines using artificial neural networks. Food Chem..

[76] Peluso I., Miglio C., Morabito G., Ioannone F., Serafini M. (2015). Flavonoids and immune function in human: A systematic review. Crit. Rev. Food Sci. Nutr..

[77] Apak R., Güçlü K., Demirata B., Özyürek M., Çelik S.E., Bektaşoğlu B., Özyurt D. (2007). Comparative evaluation of various total antioxidant capacity assays applied to phenolic compounds with the CUPRAC assay. Molecules.

[78] Huang D., Ou B., Prior R.L. (2005). The Chemistry behind Antioxidant Capacity Assays. J. Agric. Food Chem..

[79] Abougazar H., Bedir E., Khan I.A., Calis I. (2003). Wiedemanniosides A-E: New phenylethanoid glycosides from the roots of *Verbascum wiedemannianum*. Planta Med..

[80] Grigorov M., Pavlović D., Zlatković B., Dragićević A., Tadić V., Matejić J., Antić S.M., Ilić D., Nešić I. (2025). Comparative Study of Three *Verbascum* Species (*V. phlomoides*, *V. niveum* and *V. speciosum*)—Chemical Characterization and Biological Activities. Nat. Prod. Commun..

[81] Fan M., Ding H., Zhang G., Hu X., Gong D. (2019). Relationships of dietary flavonoid structure with its tyrosinase inhibitory activity and affinity. LWT.

[82] EDQM (2005). Determination of Essential Oils in Vegetable Drugs.

[83] Özek G., Özbek M., Yur S., Göger F., Arslan M., Özek T. (2019). Assessment of Endemic Cota fulvida (Asteraceae) for Phytochemical Composition and Inhibitory Activities against Oxidation, alpha-Amylase, Lipoxygenase, Xanthine Oxidase and Tyrosinase Enzymes. Rec. Nat. Prod..

[84] Singleton V.L., Orthofer R., Lamuela-Raventos R.M. (1999). Analysis of total phenols and other oxidation substrates and antioxidants by means of FolinCiocalteu reagent. Methods Enzymol..

[85] Miliauskas G., Venskutonis P., Van Beek T. (2004). Screening of radical scavenging activity of some medicinal and aromatic plant extracts. Food Chem..

[86] El Rayess Y., Barbar R., Wilson E.A., Bouajila J. (2014). Analytical Methods for Wine Polyphenols Analysis and or Their Antioxidant Activity Evaluation, Wine: Phenolic Composition, Classification and Health Benefits.

[87] Papandreou M.A., Kanakis C.D., Polissiou M.G., Efthimiopoulos S., Cordopatis P., Margarity M., Lamari F.N. (2006). Inhibitory activity on amyloid-β aggregation and antioxidant properties of *Crocus sativus* stigmas extract and its crocin constituents. J. Agric. Food Chem..

[88] Obón J.M., Castellar M.R., Cascales J.A., Fernández-López J.A. (2005). Assessment of the TEAC method for determining the antioxidant capacity of synthetic red food colorants. Food Res. Int..

[89] Masuda T., Yamashita D., Takeda Y., Yonemori S. (2005). Screening for tyrosinase inhibitors among extracts of seashore plants and identification of potent inhibitors from *Garcinia subelliptica*. Biosci. Biotechnol. Biochem..

[90] Ismaya W.T., Rozeboom H.J., Weijn A., Mes J.J., Fusetti F., Wichers H.J., Dijkstra B.W. (2011). Crystal structure of *Agaricus bisporus* mushroom tyrosinase: Identity of the tetramer subunits and interaction with tropolone. Biochemistry.

