# Supplementary material for: Phytochemical Characterization and Evaluation of Antioxidant and Tyrosinase Inhibitory Activities of Verbascum wiedemannianum Essential Oil and Methanolic Extract
Source: Molecules. 2026 May 22;31(11):1783. doi: 10.3390/molecules31111783 (PMC13257683; doi:10.3390/molecules31111783)
Supplement: Supplementary file 1 [file molecules-31-01783-s001.zip › Verbascum wiedemannianum spectrums.pdf]

Verbascum wiedemannianum LC-MS/MS Spectrums

LC-MS/MS analysis were performed using a Absciex 3200 Q trap MS/MS dedector. Experiments were performed with a Shimadzu 20A HPLC system coupled to an Applied Biosystems 3200 Q-Trap LC- MS/MS instrument equipped with a ESI ion source was used in the negative ionization mode. Separations were performed on a GL Science Intersil ODS 250 x 4,6 mm, i.d., 5 µm particle size, octadecyl silica gel analytical column operating at 40º C at a flow rate of 0.7 mL/min. Detection was carried out with PDA detector. Elution was carried out using a binary gradient of the solvent mixture Acetonitrile:Water:Formic acid (10:89:1, v/v/v) (solvent A) and Acetonitrile:Water:Formic acid (89:10:1, v/v/v) (solvent B). The composition of B was increased from 10% to 100% in 40 min. For data acquisition and analysis Analyst 1.6 software was used. For enhanced mass scan (EMS), the MS was operated at mass range of 100-1000 amu. Enhanced product ion spectra were measured from m/z 100 up to m/z1000. Nitro-gen used as the collision gas, and the collision energy was set at 30. The parameters were as follows: Collusion Energy Spread (CES)-0, Declustiring Potential (DP)-20, Enterance Potential (EP)-10, Curtain gas (CUR)-20, Gas Source 1 (GS1)-50, Gas Source 2 (GS2)-50, CAD- medium, Ihe- on and Temperature (TEM)-600. For the IDA experiment, the criteria were arranged for ions greater than 100.000 m/z and smaller than 1000 m/z and excluded former target ions after 3.0 occurrence(s) for 3.000 seconds.

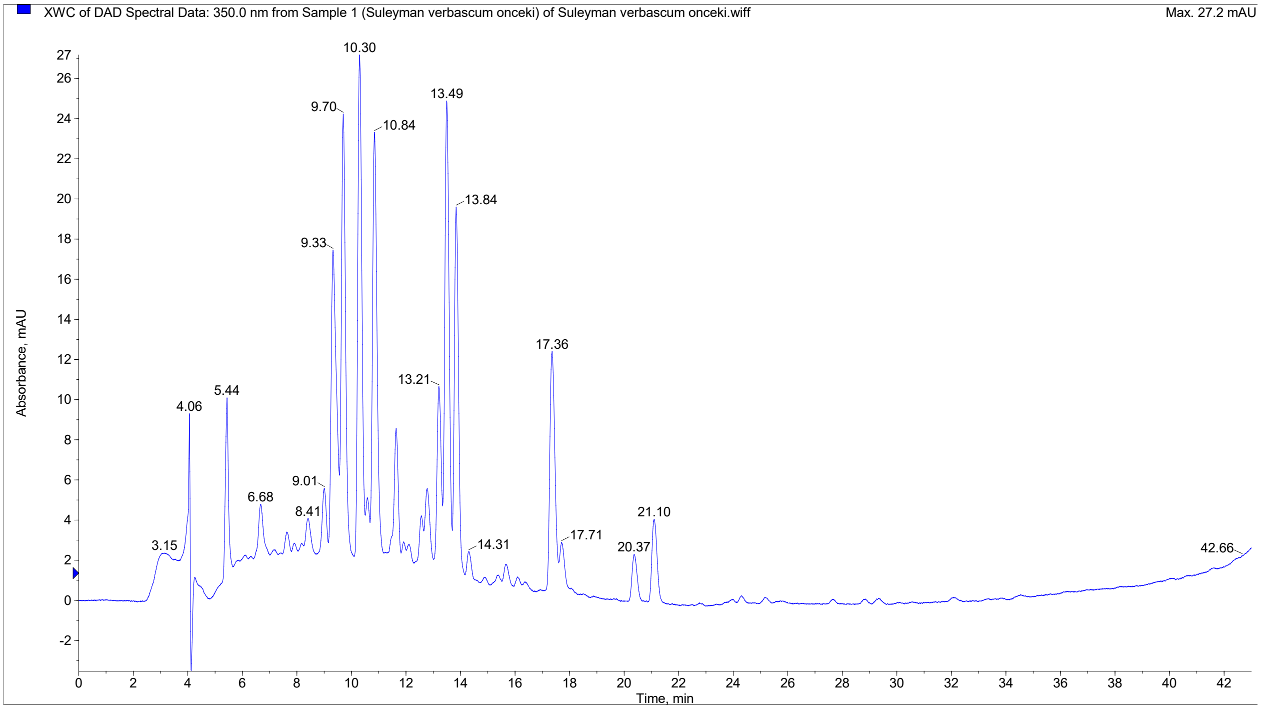

350nm LC chromatogram of the verbascum extract

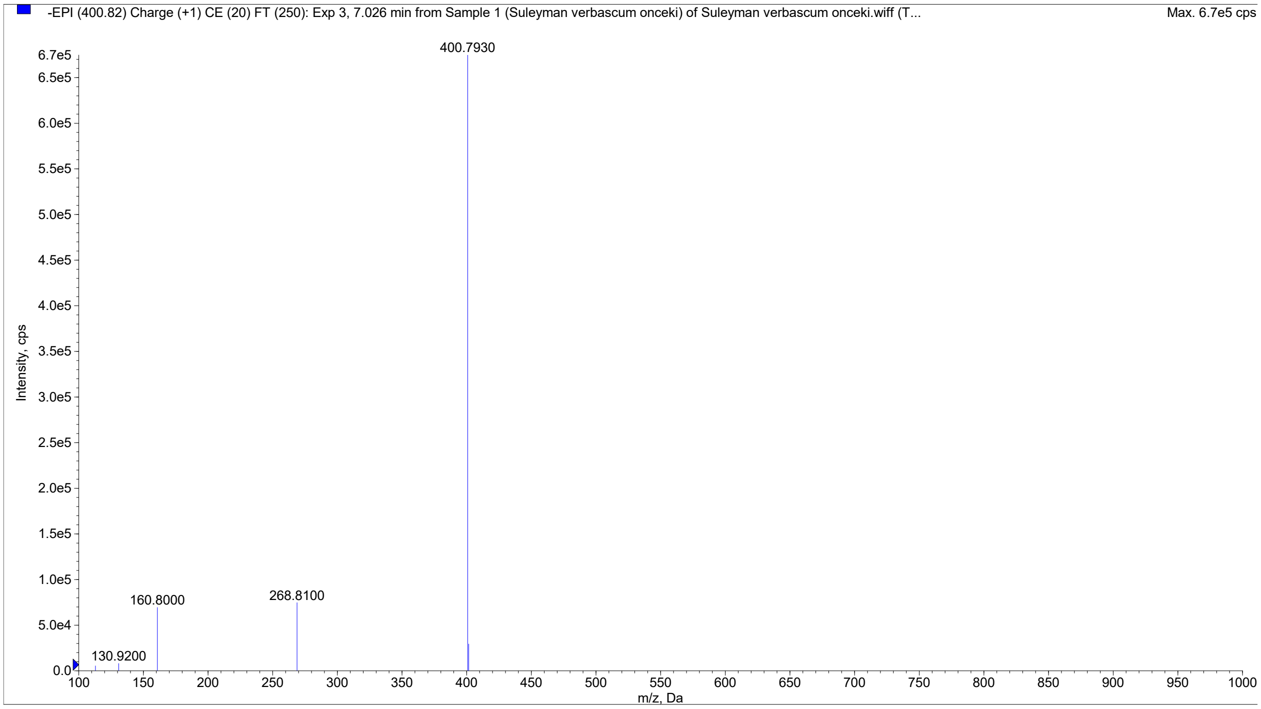

Apigenin pentoside

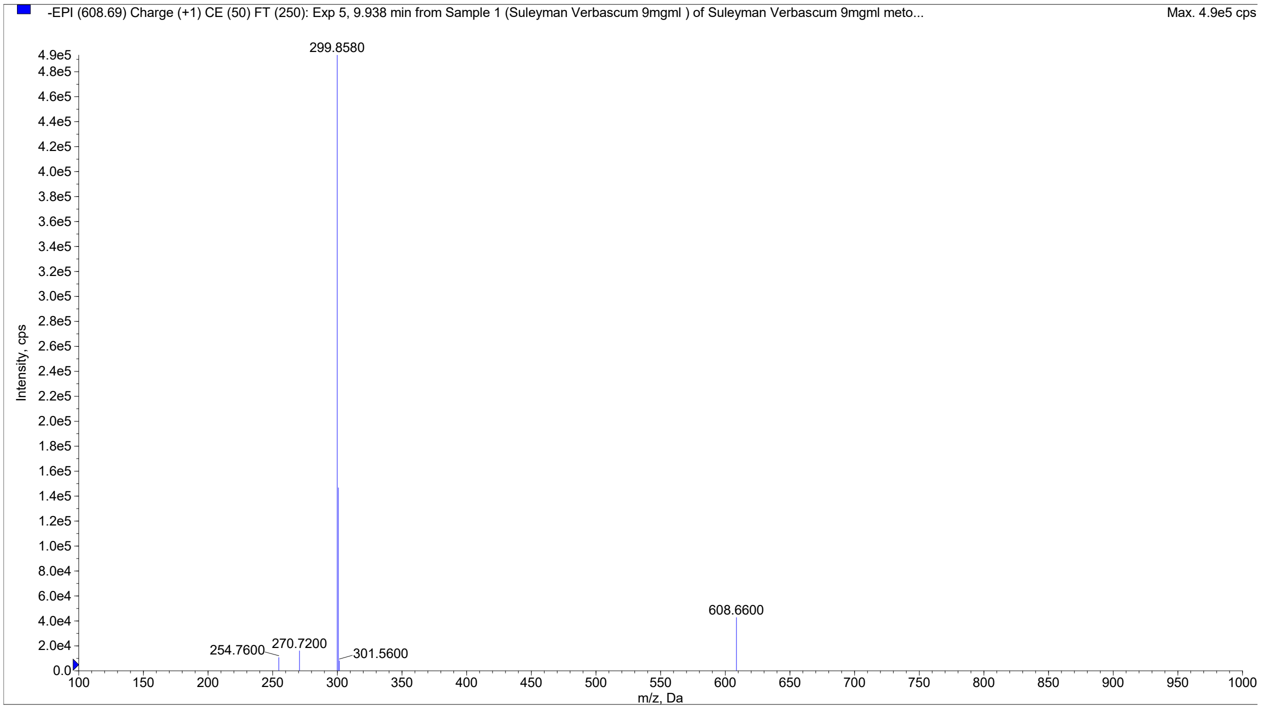

Rutin spectrum CE 50EV

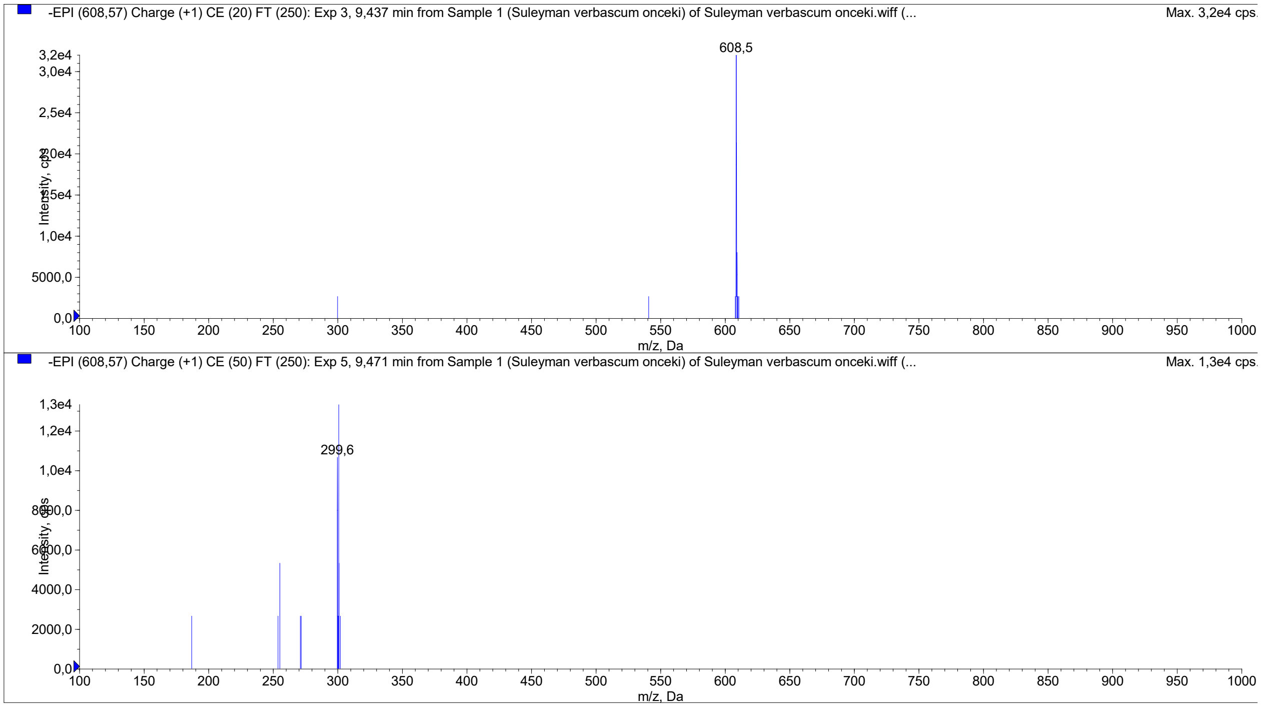

Rutin spectrum CE 20EV

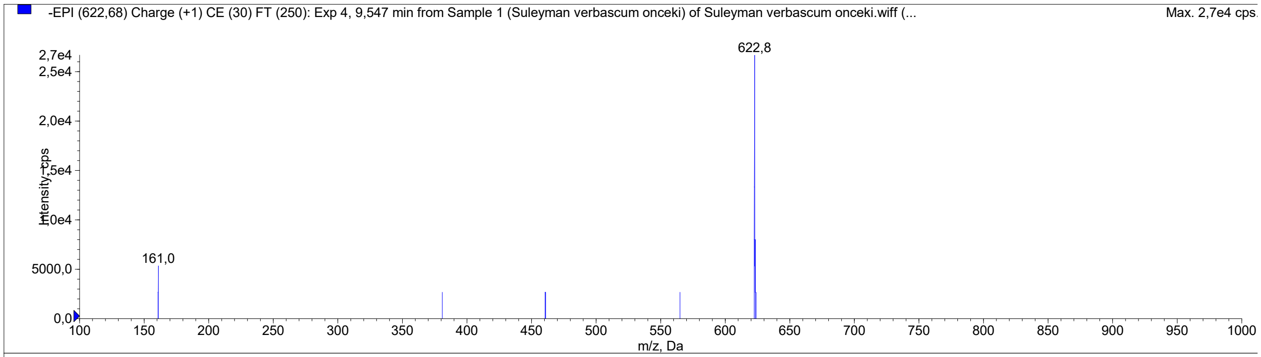

Data List for " -EPI (622,68) Charge (+1) CE (30) FT (250): Exp 4, 9,547 min from Sample 1 (Suleyman verbascum onceki) of Suleyman verbascum onceki.wiff (Turbo Spray)"

	m/z (Da)	Intensity (cps)	% Max. Intensity
1	160,8000	2666,6667	10,0000
2	160,9200	2666,6667	10,0000
3	161,0400	5333,3335	20,0000
4	380,8800	2666,6667	10,0000
5	460,5600	2666,6667	10,0000
6	460,6800	2666,6667	10,0000
7	460,9200	2666,6667	10,0000
8	564,9600	2666,6667	10,0000
9	622,4400	8000,0000	30,0000
10	622,5600	1,3333e4	50,0000
11	622,6800	1,8667e4	70,0000
12	622,8000	2,6667e4	100,0000
13	622,9200	5333,3335	20,0000
14	623,0400	8000,0000	30,0000

Verbascoside

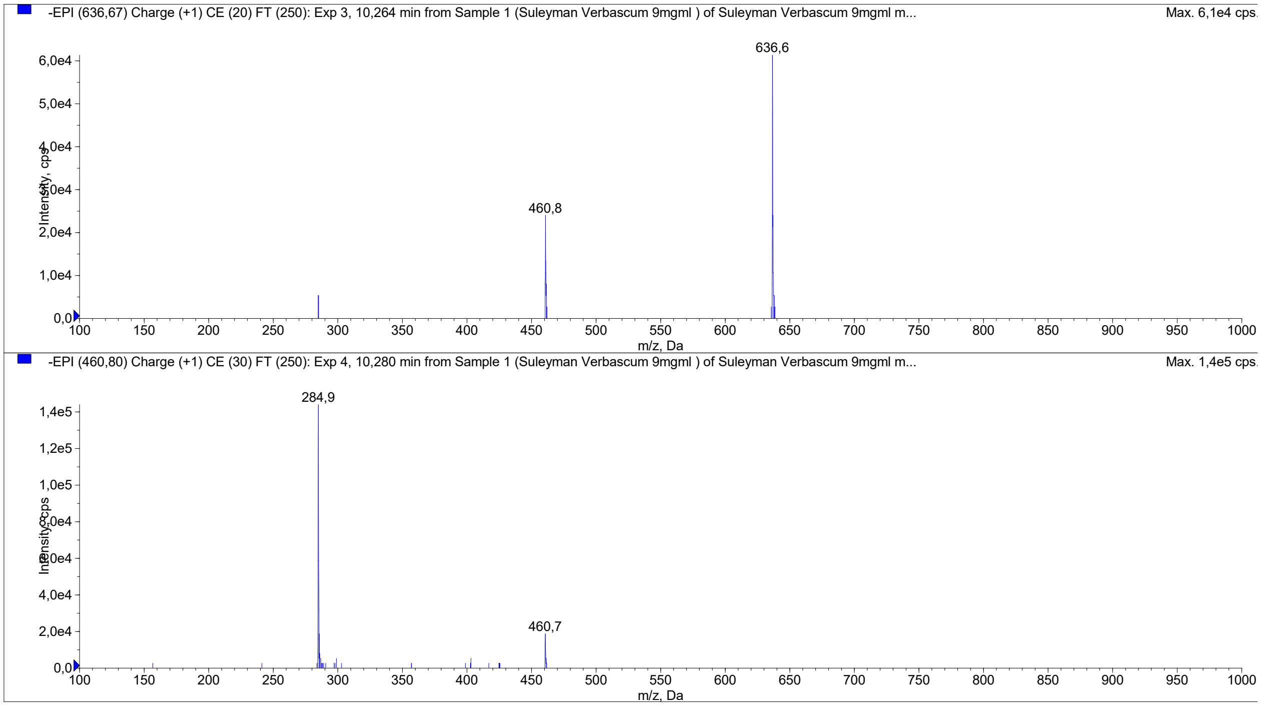

Luteolin diglucuronide CE 20, 30EV

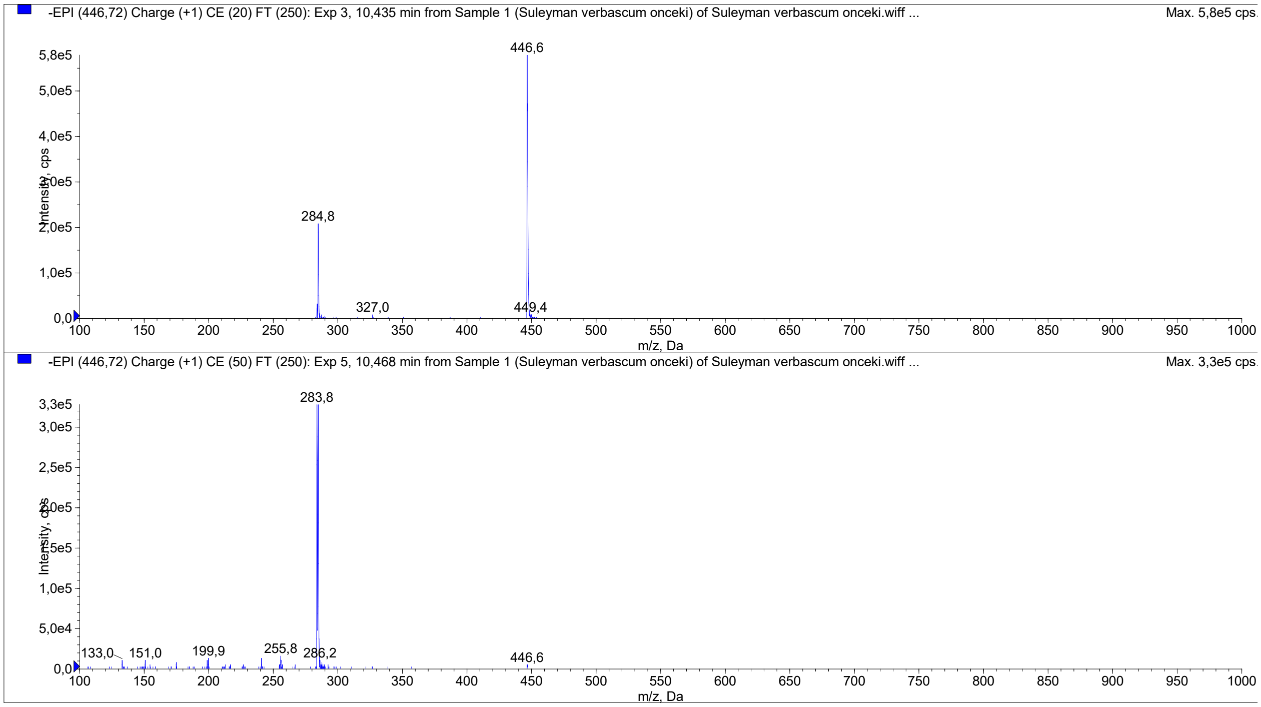

Luteolin glucoside

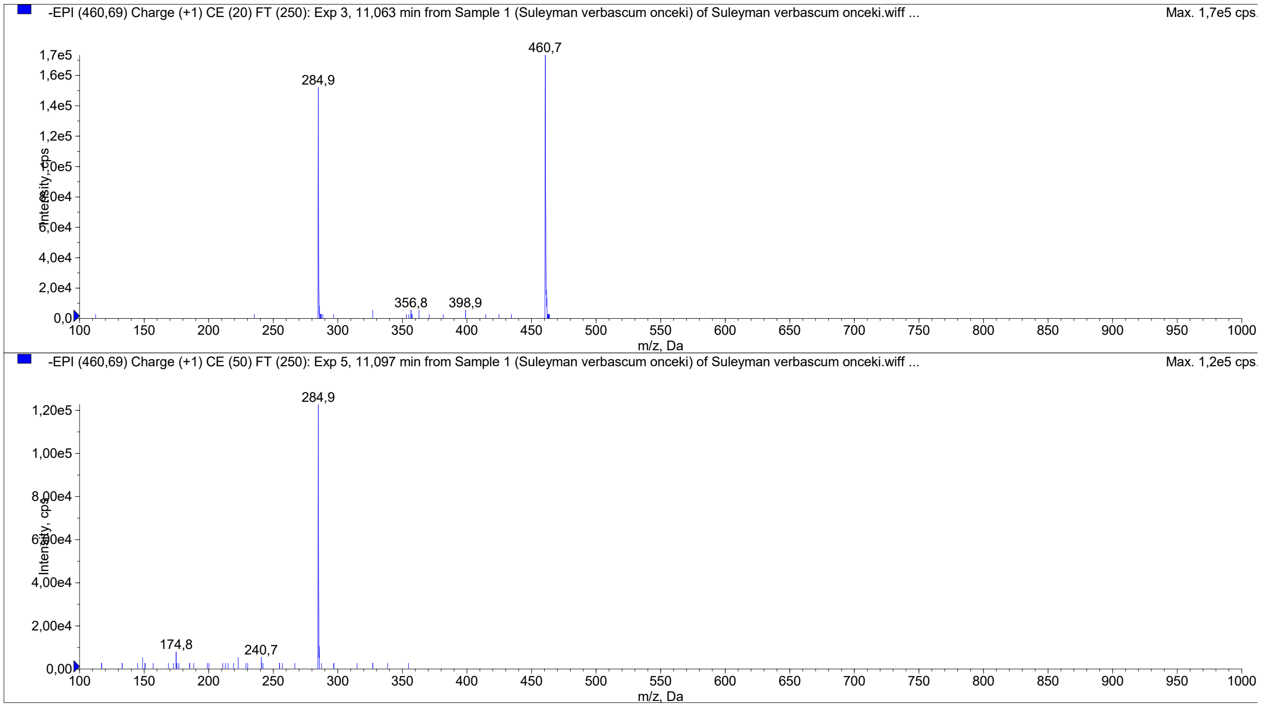

Luteolin glucuronide at Rt 11

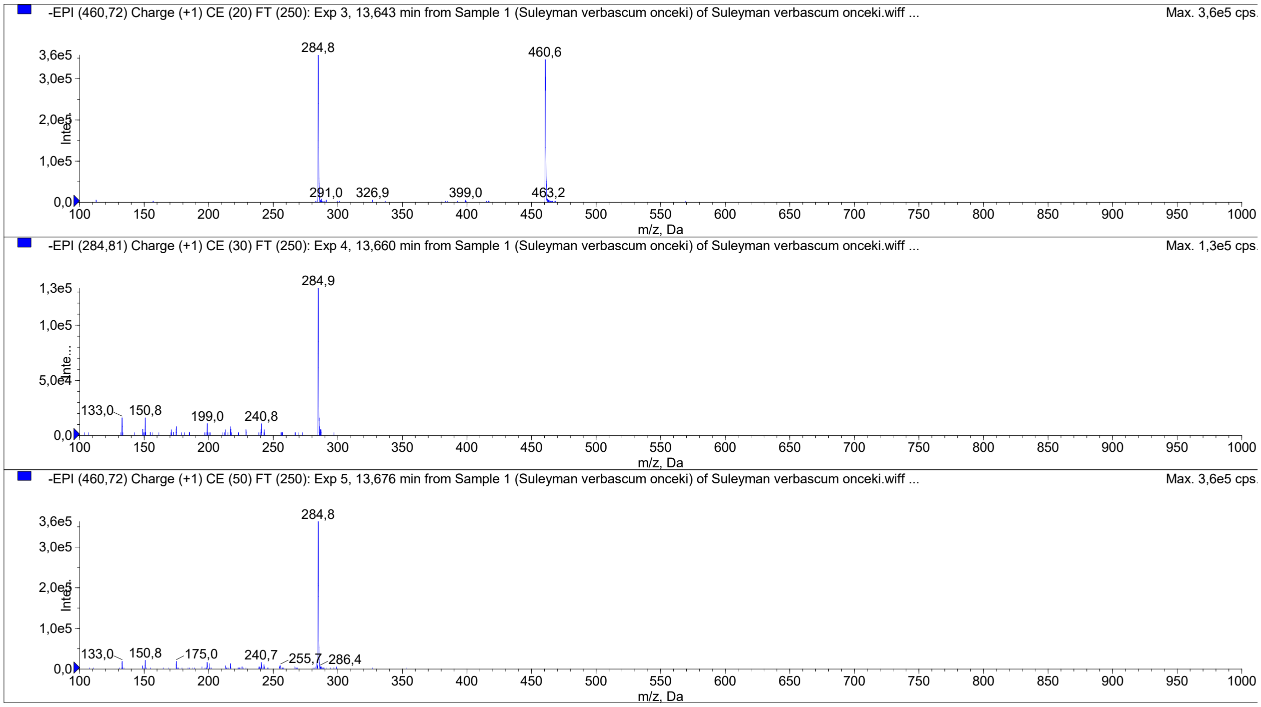

Luteolin glucuronide at Rt 13

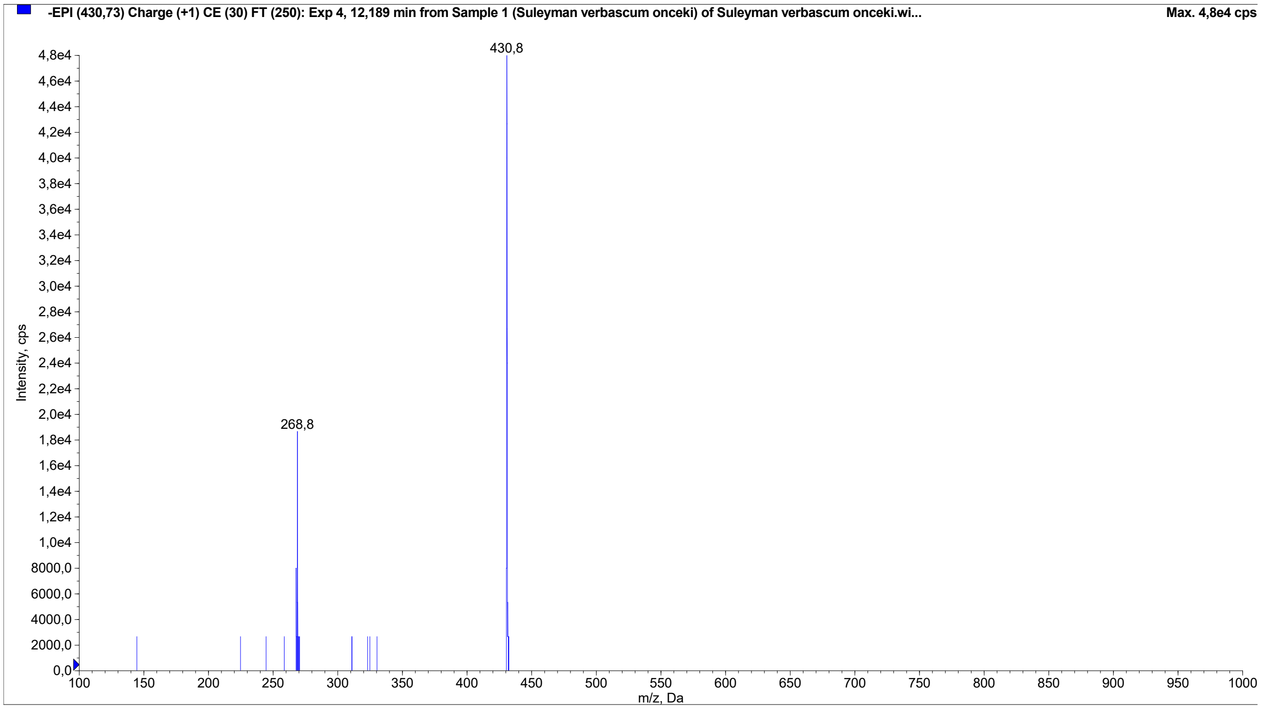

Apigenin glucoside

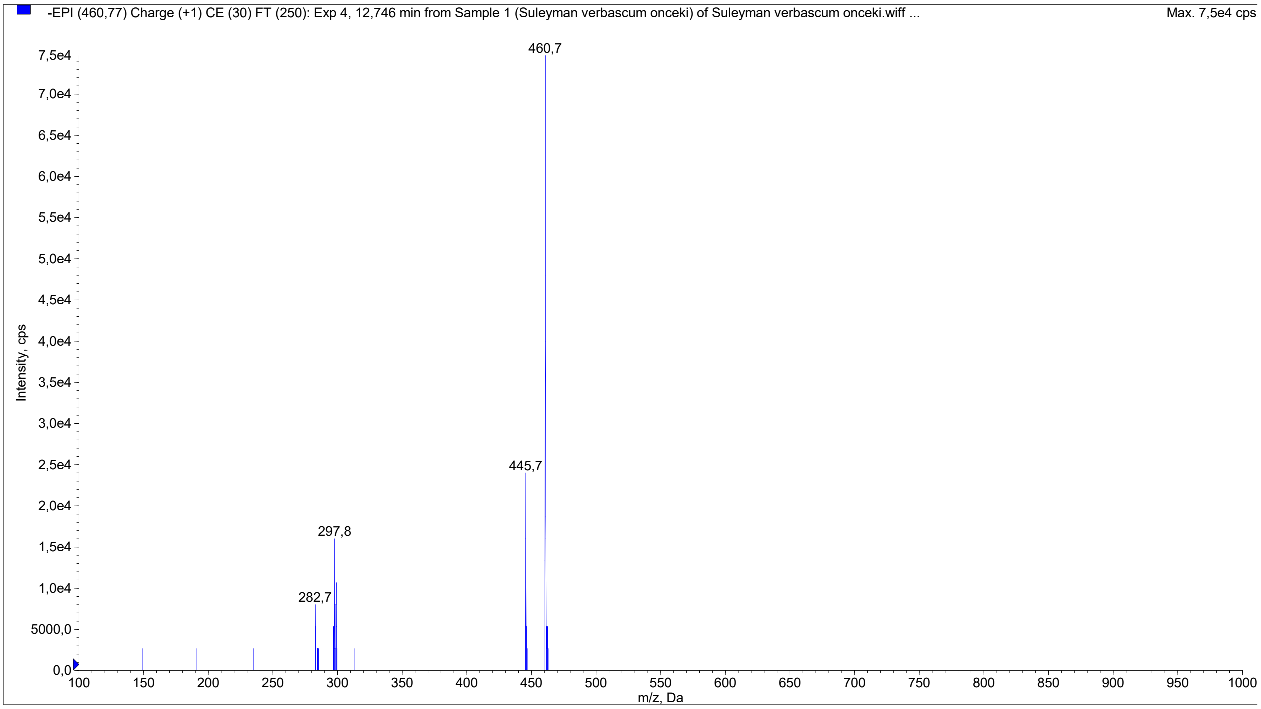

Chrysoeriol glucoside

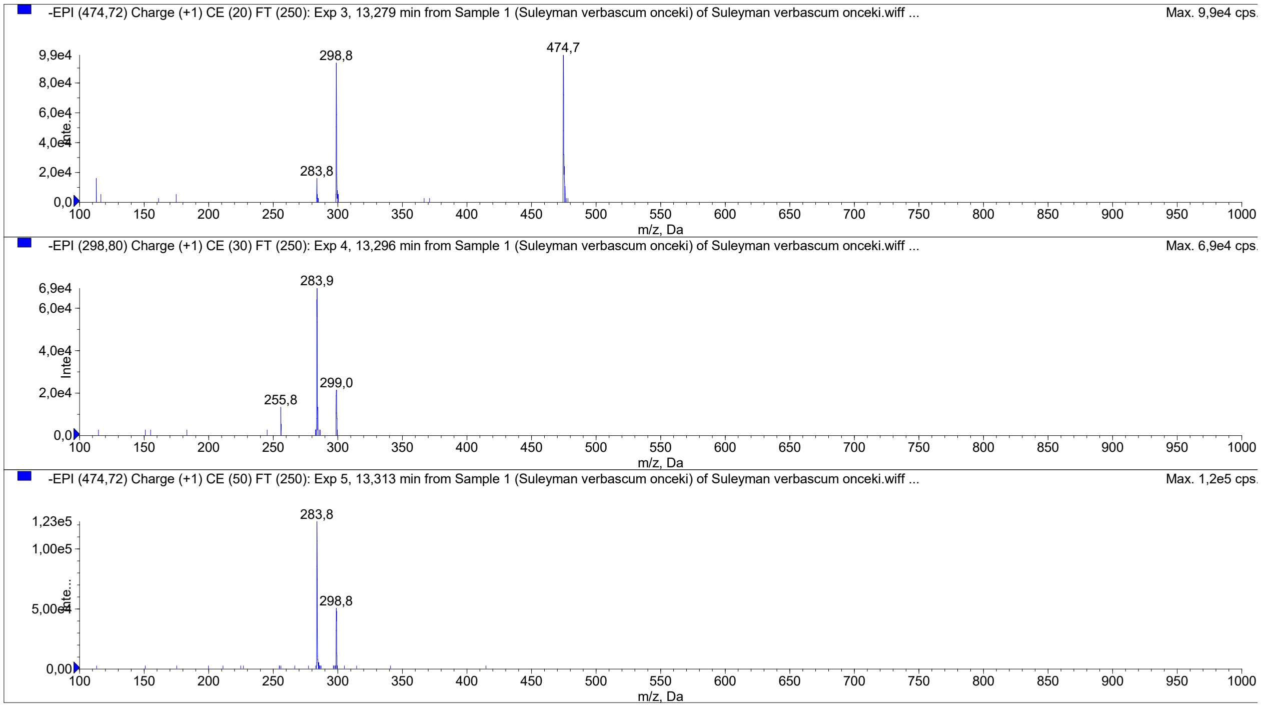

Chrysoeriol glucuronide

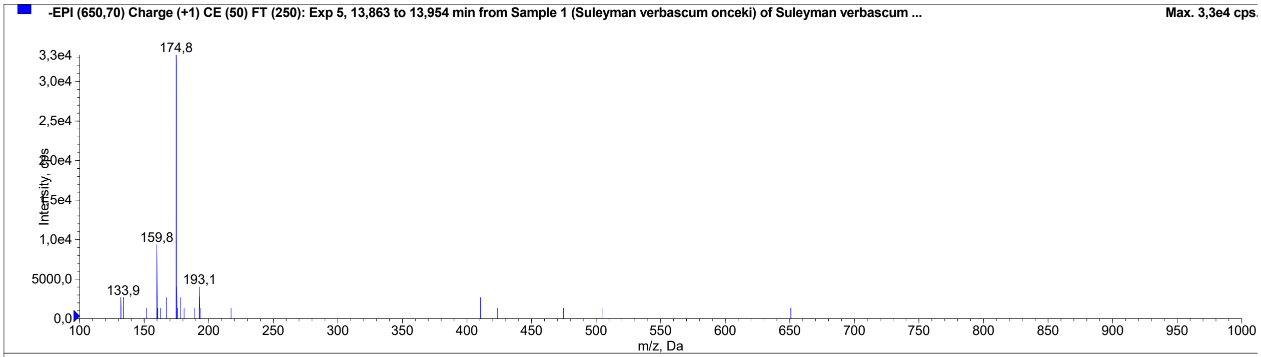

Data List for " -EPI (650,70) Charge (+1) CE (50) FT (250): Exp 5, 13,863 to 13,954 min from Sample 1 (Suleyman verbascum onceki) of Suleyman verbascum onceki.wiff (Turbo Spray)"

	m/z (Da)	Intensity (cps)	% Max. Intensity
1	131,8800	2666,6667	8,0000
2	132,0000	2666,6667	8,0000
3	132,1200	1333,3334	4,0000
4	133,8000	1333,3334	4,0000
5	133,9200	2666,6667	8,0000
6	151,8000	1333,3334	4,0000
7	159,7200	2666,6667	8,0000
8	159,8400	9333,3334	28,0000
9	159,9600	6666,6669	20,0000
10	160,0800	2666,6667	8,0000
11	160,4400	1333,3334	4,0000
12	160,5600	1333,3334	4,0000
13	162,6000	1333,3334	4,0000
14	167,1600	2666,6667	8,0000

Martynoside

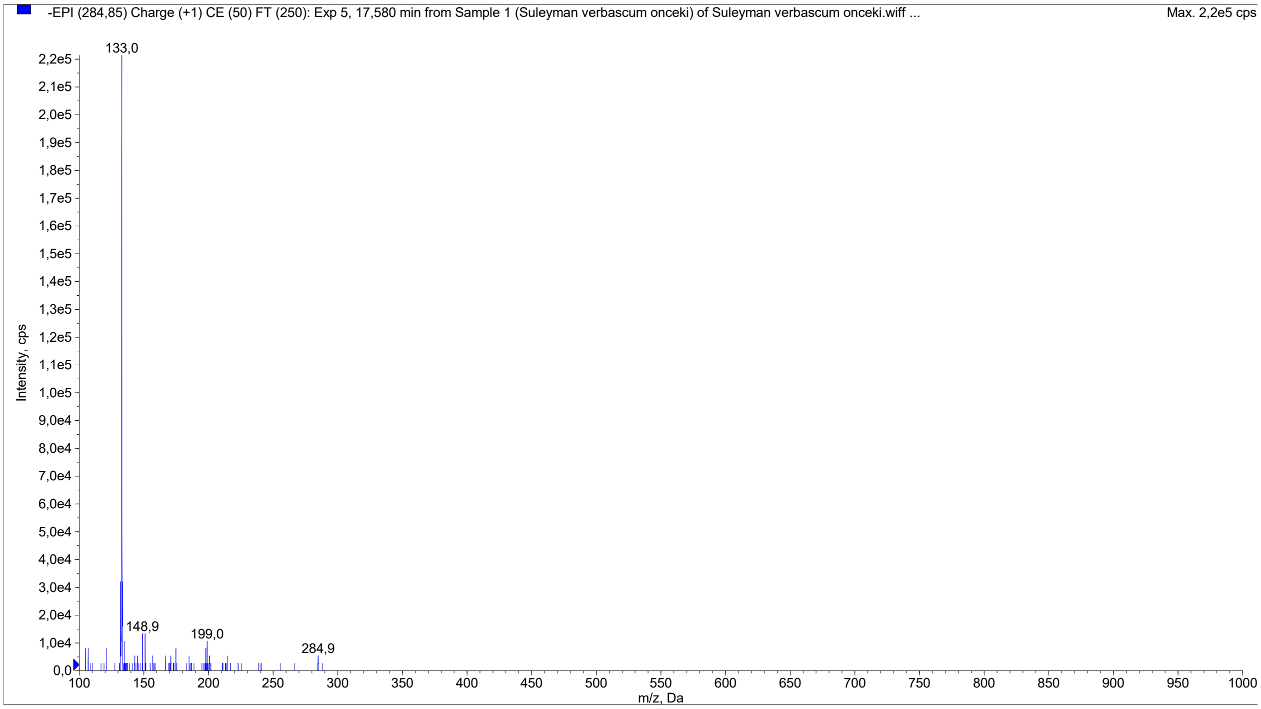
-EPI (284,85) Charge (+1) CE (50) FT (250): Exp 5, 17,580 min from Sample 1 (Suleyman verbascum onceki) of Suleyman verbascum onceki.wiff ...
Max. 2,2e5 cps
133,0
148,9
199,0
284,9
Intensity, cps
m/z, Da

Luteolin

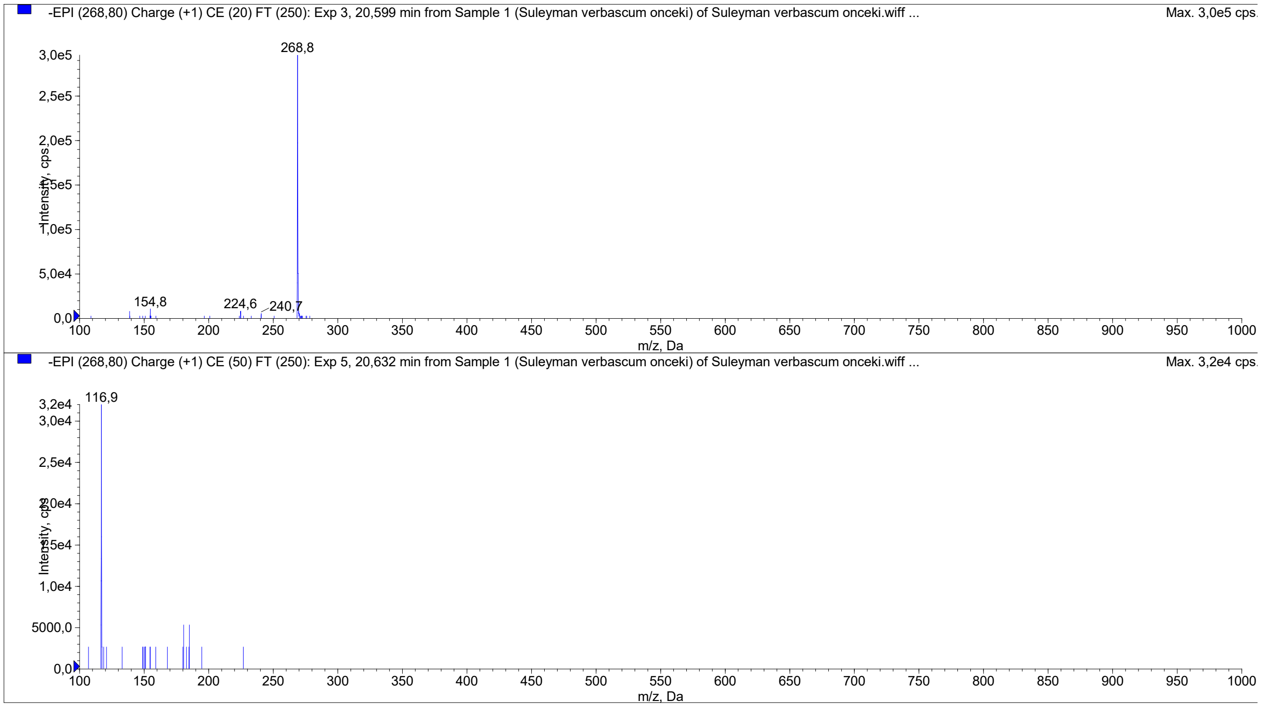
-EPI (268,80) Charge (+1) CE (20) FT (250): Exp 3, 20,599 min from Sample 1 (Suleyman verbascum onceki) of Suleyman verbascum onceki.wiff ...
Max. 3,0e5 cps.
268,8
154,8
224,6
240,7
Intensity, cps
m/z, Da
-EPI (268,80) Charge (+1) CE (50) FT (250): Exp 5, 20,632 min from Sample 1 (Suleyman verbascum onceki) of Suleyman verbascum onceki.wiff ...
Max. 3,2e4 cps.
116,9
Intensity, cps
m/z, Da

Apigenin

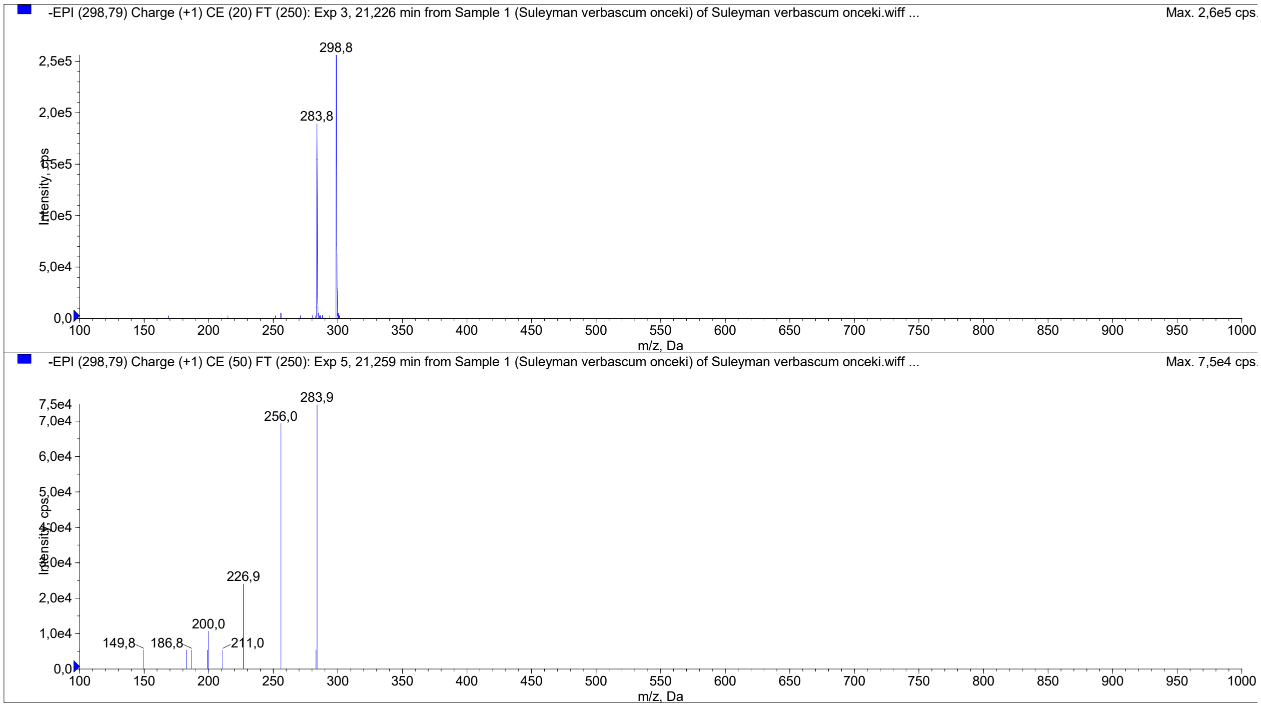

Chrysoeriol
