# Supplementary material for: Phytochemical Characterization and Evaluation of Antioxidant and Tyrosinase Inhibitory Activities of Verbascum wiedemannianum Essential Oil and Methanolic Extract
Source: Molecules. 2026 May 22;31(11):1783. doi: 10.3390/molecules31111783 (PMC13257683; doi:10.3390/molecules31111783)
Supplement: Supplementary file 1 [file molecules-31-01783-s001.zip › Enago-Certificate-GYSCAW-106 verbascum Enago sertifika.pdf]

Author First, Quality First

CERTIFICATE OF EDITING

This document certifies that the manuscript

Phytochemical Characterization and Evaluation of Antioxidant and Tyrosinase Inhibitory Activities of Verbascum wiedemannianum

Prepared by the Author(s)

Dr. Fatih Goger

was edited for English language, grammar, punctuation, spelling and style by one or more highly qualified English speaking editors.

This certificate was issued on

22 Apr 2026

THE ENAGO PROMISE Quality Guarantee

Michelle Miller

Operations Director - Enago

Crimson Interactive Inc.
Delaware, 19904, USA

Since 2005, Enago has supported over 2 million research authors from 125+ countries in preparing their manuscripts for publication in international peer-reviewed journals. With a team of 3000+ experts, we offer professional academic editing to ensure the highest standards of language and grammar accuracy.

ISO/IEC 27001 : 2022 Information Security Management Systems

ISO 9001:2015 Quality Management Systems

Disclaimer: The intent of the author's message has been preserved during the editing process. The author is free to accept or reject our changes in the manuscript after reviewing our edits. This certificate has been awarded at the time of sharing the final edited version with the author. Enago does not bear any responsibility for any alterations done by the author to the edited document post date of issue, 22 Apr 2026.
