# Supplementary figures and images for: Phytochemical Characterization and Evaluation of Antioxidant and Tyrosinase Inhibitory Activities of Verbascum wiedemannianum Essential Oil and Methanolic Extract
Source: Molecules. 2026 May 22;31(11):1783. doi: 10.3390/molecules31111783 (PMC13257683; doi:10.3390/molecules31111783)

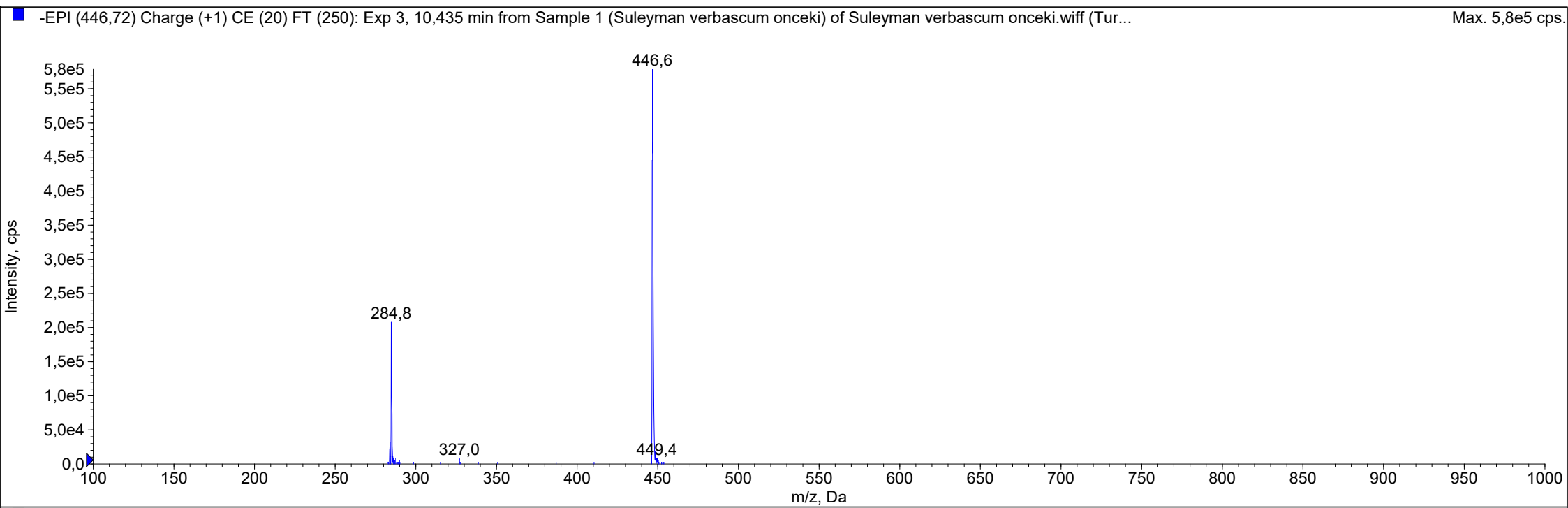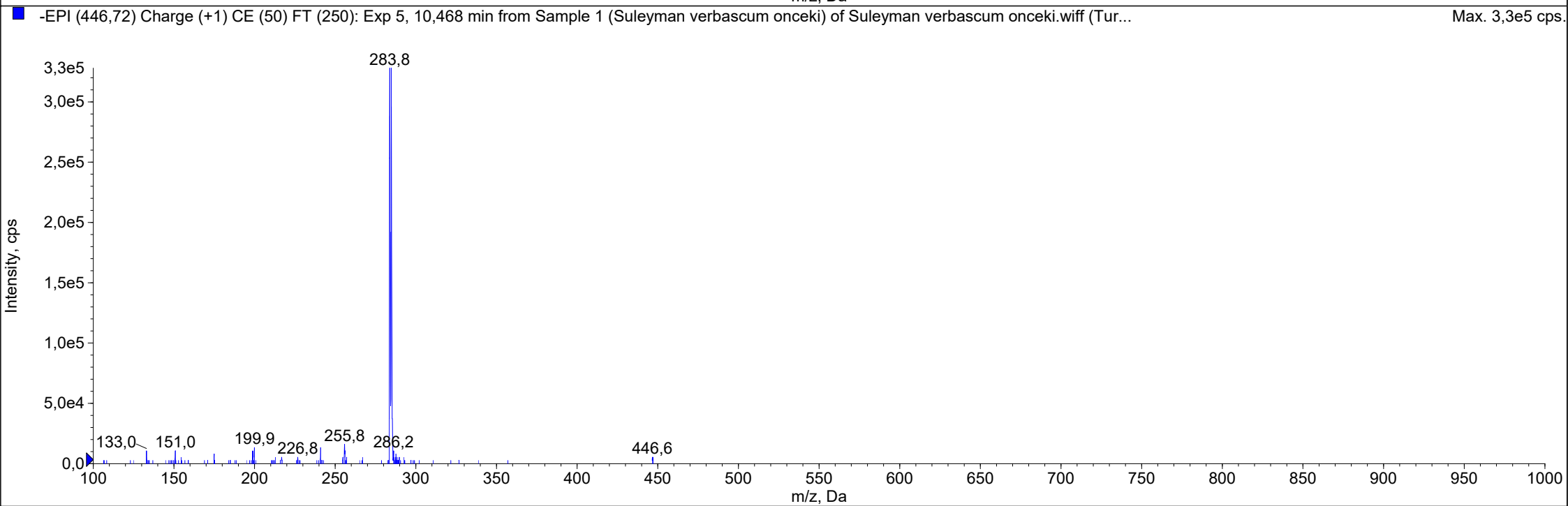

Supplement: Supplementary file 1 [file molecules-31-01783-s001.zip › Luteolin glucoside mass spectrum.pdf]

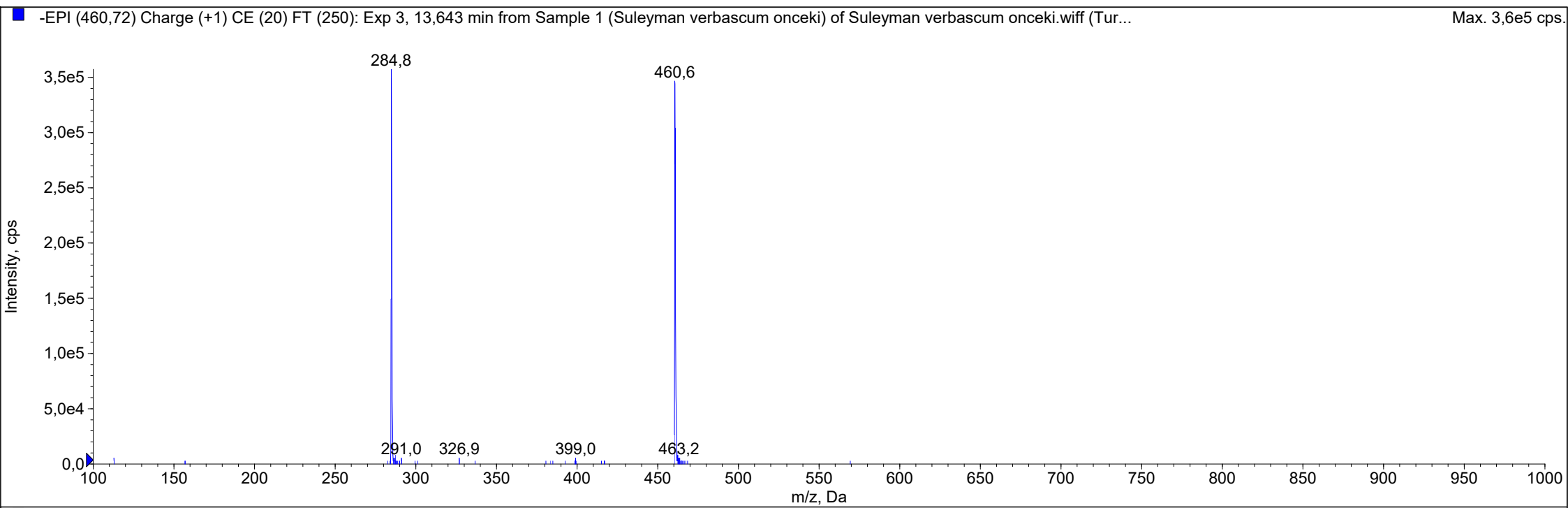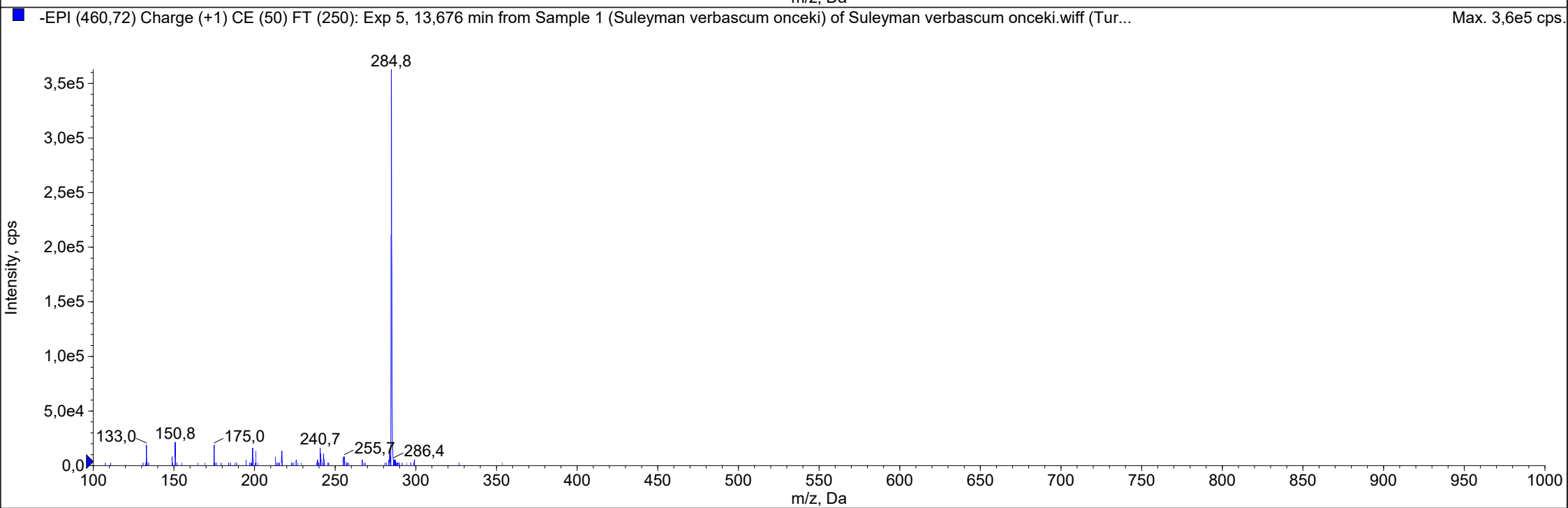

Supplement: Supplementary file 1 [file molecules-31-01783-s001.zip › luteolin glucuronide mass spectrum.pdf]

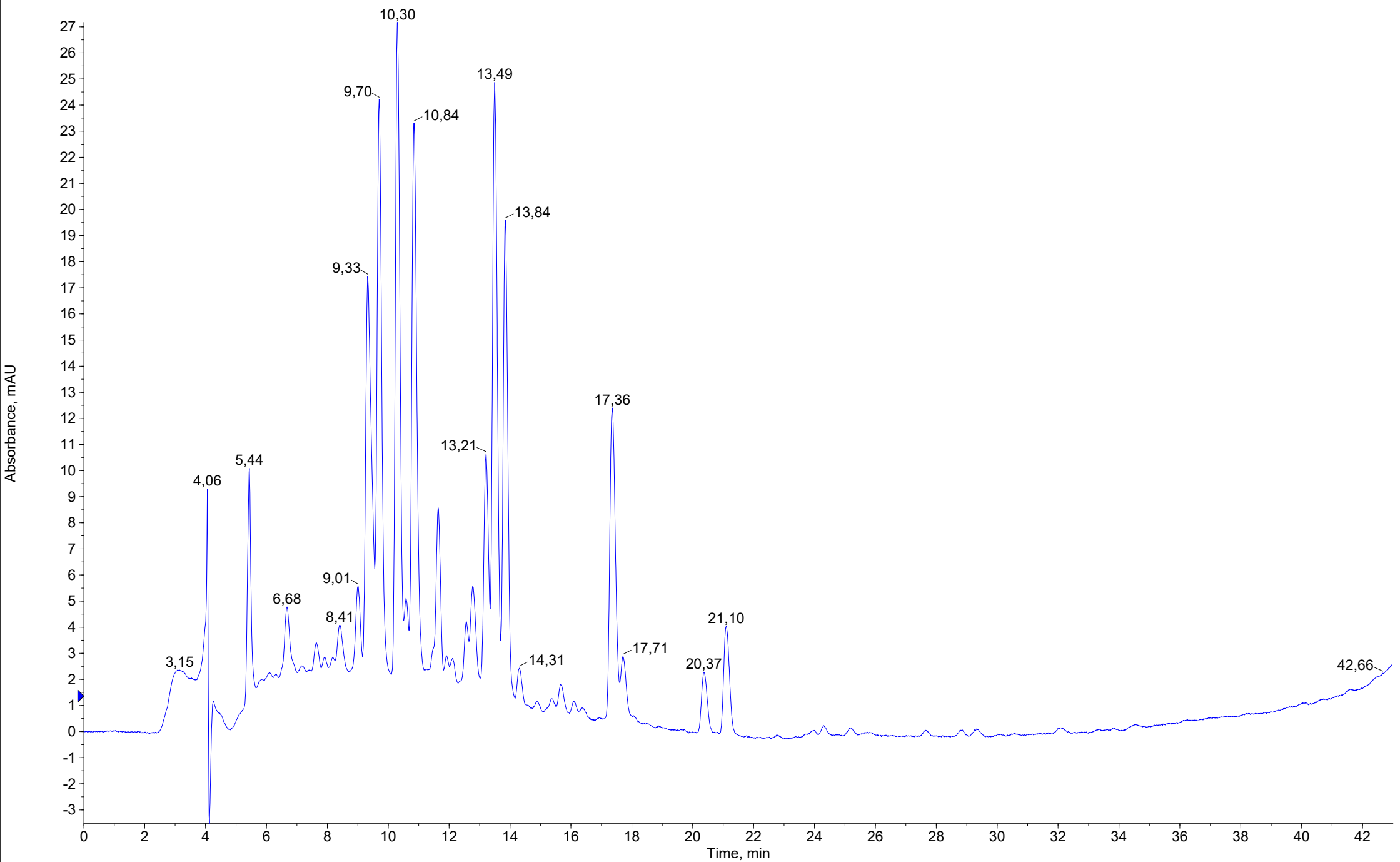

Supplement: Supplementary file 1 [file molecules-31-01783-s001.zip › Vw. 350nm.pdf]

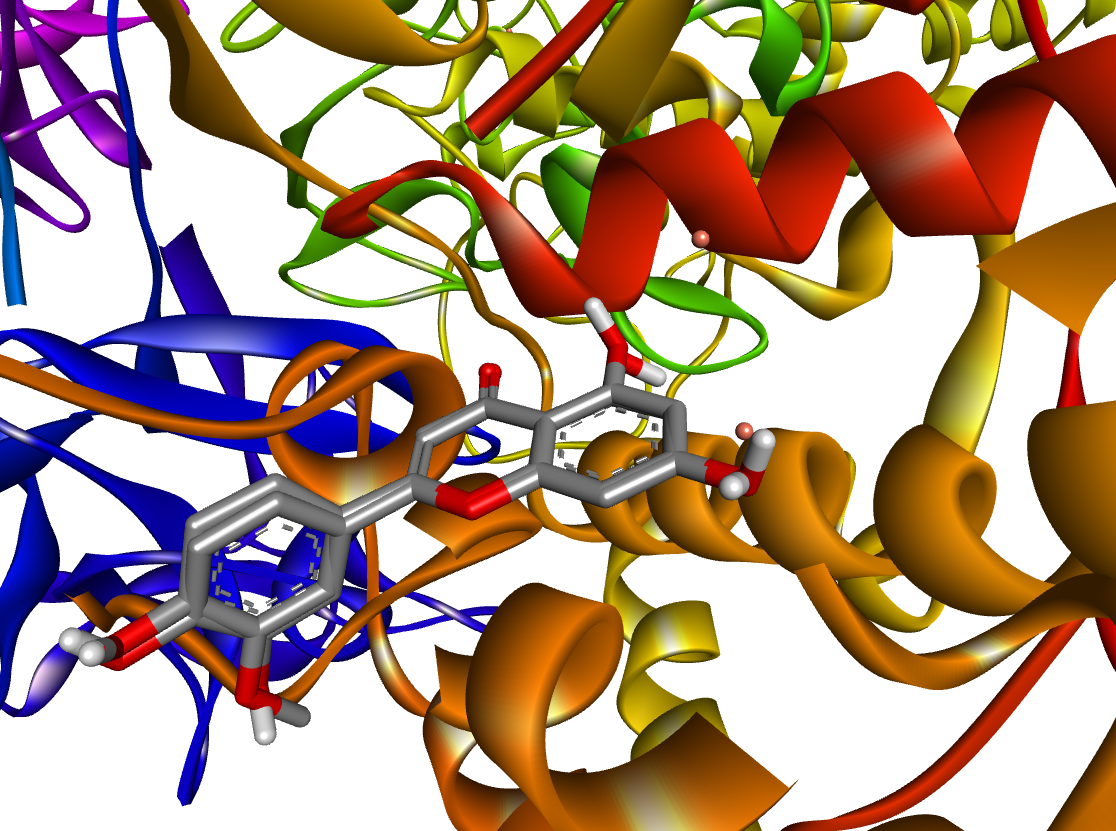

Supplement: Supplementary file 1 [file molecules-31-01783-s001.zip › 2Y9X_CHRYSERIOL&LUTEOLİN_ACTIVESITE.png]

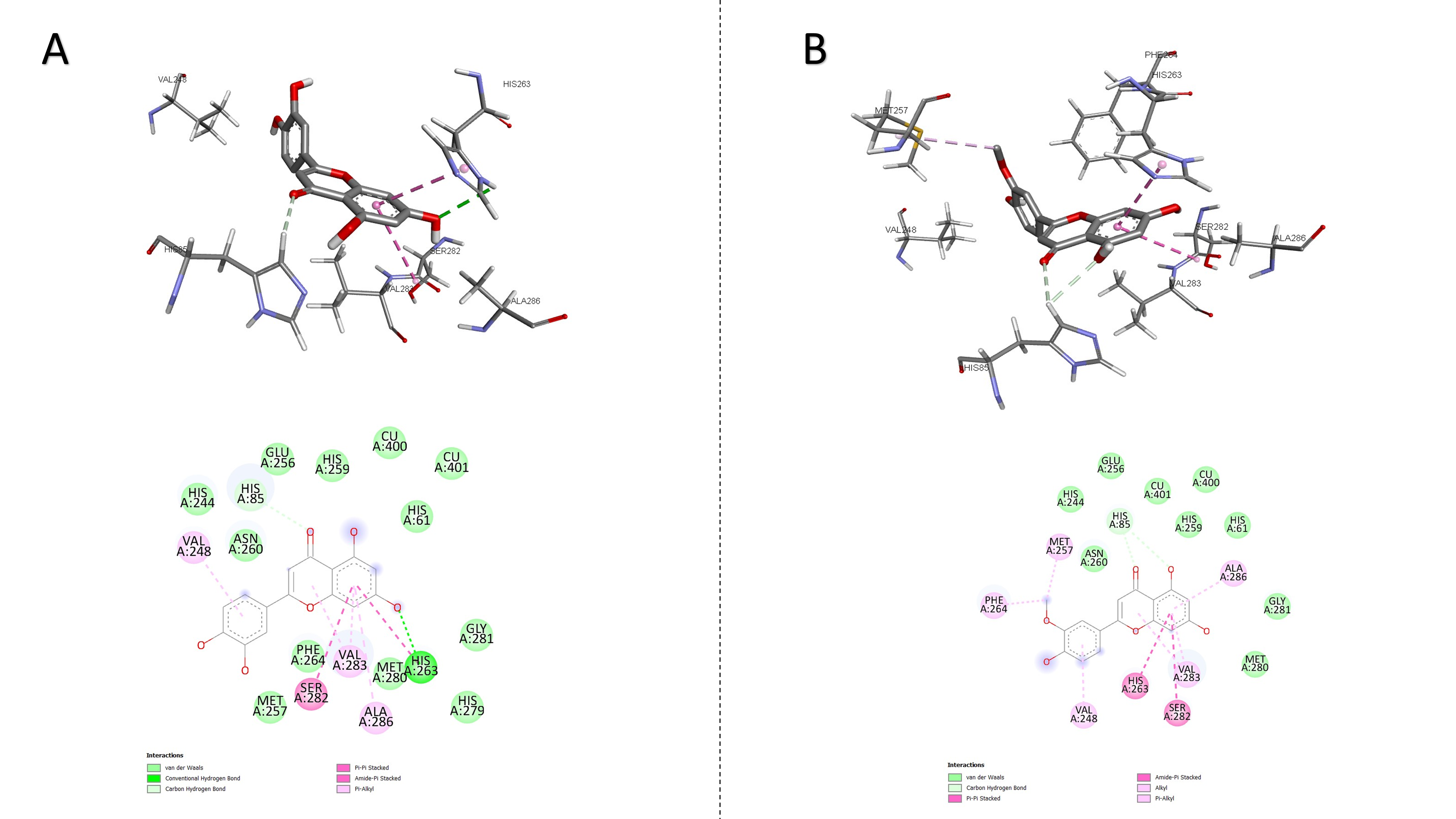

Supplement: Supplementary file 1 [file molecules-31-01783-s001.zip › 3D&2D interactions of luteolin and chryseriol.png]
